# Ancient DNA reveals kinship burial patterns of a pre-Columbian Andean community

**DOI:** 10.1186/1471-2156-13-30

**Published:** 2012-04-23

**Authors:** Mateusz Baca, Karolina Doan, Maciej Sobczyk, Anna Stankovic, Piotr Węgleński

**Affiliations:** 1 Center for Precolumbian Studies, University of Warsaw, Krakowskie Przedmieście 26/28, 00-927, Warsaw, Poland; 2 Institute of Genetics and Biotechnology, Department of Biology, University of Warsaw, Pawińskiego 5A, 02–106, Warsaw, Poland; 3 Institute of Biochemistry and Biophysics, Polish Academy of Science, Pawińskiego 5A, 02-106, Warsaw, Poland; 4 Centre of New Technologies, University of Warsaw, Miecznikowa 1, 02-096, Warsaw, Poland

## Abstract

**Background:**

A detailed genetic study of the pre-Columbian population inhabiting the Tompullo 2 archaeological site (department Arequipa, Peru) was undertaken to resolve the kin relationships between individuals buried in six different chullpas. Kin relationships were an important factor shaping the social organization in the pre-Columbian Andean communities, centering on the ayllu, a group of relatives that shared a common land and responsibilities. The aim of this study was to evaluate whether this Andean model of a social organization had an influence on mortuary practices, in particular to determine whether chullpas served as family graves.

**Results:**

The remains of forty-one individuals were analyzed with both uniparental (mtDNA, Y–chromosome) and biparental (autosomal microsatellites) markers. Reproducible HVRI sequences, autosomal and Y chromosomal STR profiles were obtained for 24, 16 and 11 individuals, respectively. Mitochondrial DNA diversity was comparable to that of ancient and contemporary Andean populations. The Tompullo 2 population exhibited the closest relationship with the modern population from the same region. A kinship analysis revealed complex pattern of relations within and between the graves. However mean relatedness coefficients regarding the pairs of individuals buried in the same grave were significantly higher than those regarding pairs buried in different graves. The Y chromosome profiles of 11 males suggest that only members of one male line were buried in the same grave.

**Conclusions:**

Genetic investigation of the population that inhabited Tompullo 2 site shows continuity between pre-Columbian and modern Native Amerindian populations inhabiting the Arequipa region. This suggests that no major demographic processes have influenced the mitochondrial DNA diversity of these populations during the past five hundred years. The kinship analysis involving uni- and biparental markers suggests that the community that inhabited the Tompullo 2 site was organized into extended family groups that were buried in different graves. This finding is in congruence with known models of social organization of Andean communities.

## Background

Ancient DNA analysis of pre-Columbian individuals from South America has most often been conducted to determine the number and timing of initial migrations to the Americas [1] and to infer demographic changes in Amerindian populations [2-6]. In these studies mitochondrial DNA (mtDNA) was used because it is usually the only marker, available for use with ancient DNA samples. More recently nuclear markers, such as microsatellites, have been successfully applied to ancient material, making detailed analysis of kinship between individuals possible [7,8].

Kin relations play an important role in pre-Columbian Andean cultures [9]. The basic sociopolitical unit of Native South Americans, the ayllu, was based on true or supposed kin relations [10-12]. The word ayllu could also refer to a group of kin relatives. The origins of ayllu-based communities are connected with the appearance of chullpas (i.e. above ground mortuary monuments) [11], which appeared in the archaeological record during the Early Intermediate Period in the northern highlands of modern Peru, and spread across the whole Central Andes, to become the predominant form of mortuary buildings in the Late Intermediate Period [11]. Apart from their funerary and religious functions, chullpa became an important element in the sociopolitical organization of Indian communities as places of collective reference, where ancestors were buried. Ancestor worship, widespread in pre-Columbian cultures [11,13], relied upon the veneration of ancestors, as shown by their presentation in public places, simulating their participation in everyday life. Thus a belief about origin from the same common ancestor was the basis for belonging to a particular group [9,11], and fundamental to ayllu formation [11,14]. Remnants of this social organization persist to the present day in the Q’ero Indians community [15].

Studies about kinship of individuals buried in different chullpas provide an extraordinary opportunity to make inferences about social organization of these groups and about the significance of the kin relation in Andean communities. Recognition of kinship between individuals based solely on the archaeological record is usually impossible, and reconstructions of kinship structure in pre-Columbian archaeological sites are therefore most often based on ethnohistorical analogies and colonial records.

In this paper we present the results of ancient DNA analysis aimed at reconstructing kin relationships of individuals buried at the Tompullo 2 site. This site, located in the vicinity of the Coropuna volcano in southern Peru, was a pastoral settlement of llama and alpaca herders. Extraordinary macroscopic preservation of human remains excavated in six chullpas at this site and environmental conditions favorable for DNA preservation made this genetic kinship analysis feasible.

## Methods

The study was conducted under the project “Condesuyos” carried out within the agreement between Warsaw University and the Universidad Católica de Santa Maria, coordinated by prof. Mariusz Ziółkowski director of Centre for Precolumbian Studies UW and dr Luis Augusto Belan Franco director of the Museo Arqueológico de la UCSM. Samples were collected under permissions granted by Ministerio de Cultura (formerly Instituto Nacional de Cultura) and stored in Museo Arqueológico de la UMCS.

### Site

**Figure 1:**
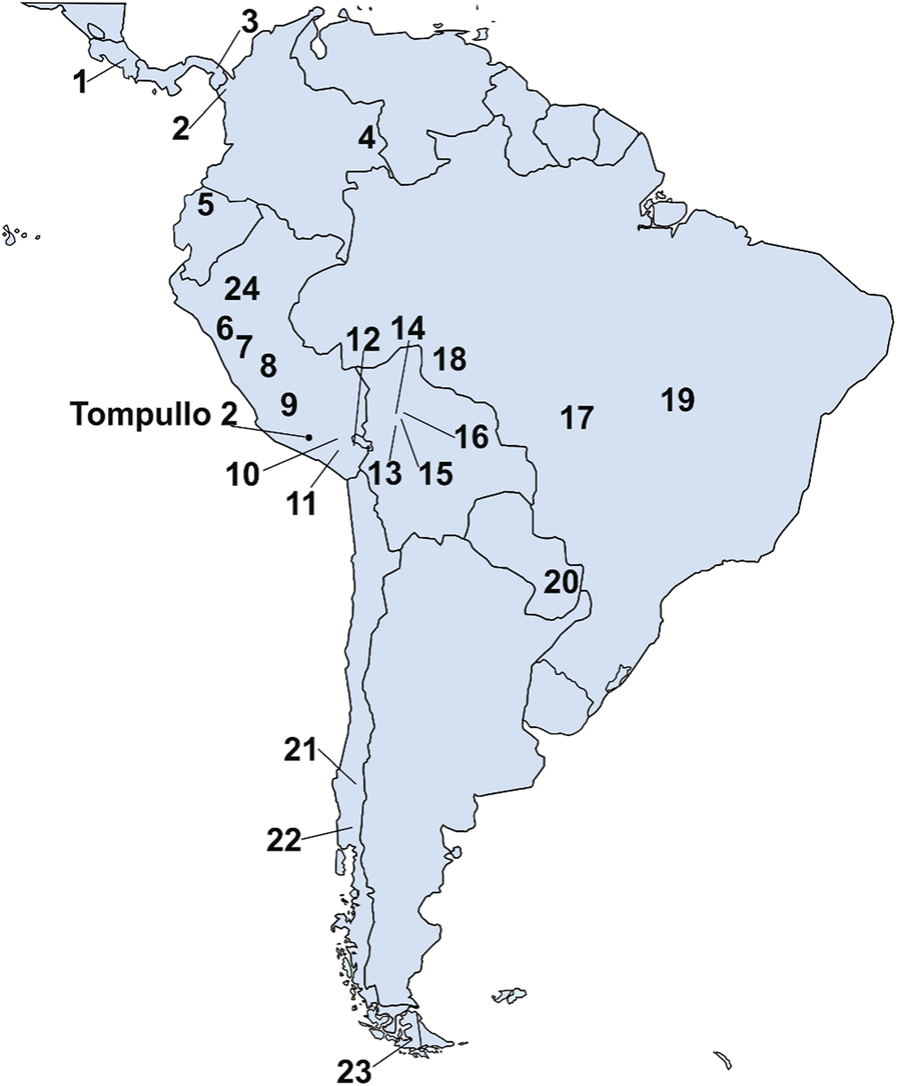
**Map of South America with location of Tompullo 2 site and contemporary populations used in this study.** 1 – Ngöebe (n = 46) [18], 2 – Embera (n = 44) [19], 3 – Wounan (n = 31) [20], 4 – Guahibo (n = 59) [21], 5 – Cayapa (n = 30) [22], 6 - Yungay ( n = 36) [23], 7 - Ancash (n = 33) [24], 8 – San Martin (n = 23) [25], 9 – Tupe (n = 16) [23], 10 – Tayacaja (n = 61) [25], 11 – Arequipa (n = 22) [25], 12- Puno (Aymara speaking) (n = 14) [23], 13 – Puno (Quechua speaking) (n = 30) [23], 14 – Yuracare (n = 15) [26], 15 - Ignaciano (n = 15) [26], 16 - Trinitario (n = 11) [26], 17 – Movima (n = 12) [26], 18 – Zoro (n = 29) [27], 19 – Gavião (n = 28) [27], 20 – Xavante (n = 24) [27], 21 – Ache (n = 63) [28], 22 – Pehuenche (n = 24) [29], 23 - Mapuche (n = 34) [29], 24 – Yaghan (n = 15) [29].

The Tompullo 2 site is located on the mountainside of Cora Cora -- about 4000 meters high, in the Andaray district (dept. Arequipa, Peru; coordinates 15°43′45”S, 72°44′50”W) (Figure 1). It was investigated as part of a project to characterize the pre-Columbian settlement pattern in the region of Coropuna volcano, led by the Centre of Precolumbian Studies of the University of Warsaw. The site stretches across approximately 5 ha, and consists of several dozen buildings. The presence of oval constructions interpreted as enclosures for llamas and/or alpacas, suggests that Tompullo 2 was a pastoral settlement. The site could probably have served as a local administration center, as indicated by the presence of public building like kallanka [16]. The Tompullo 2 site was occupied for a short period of time in the Late Horizon (15^th^--16^th^ century). It is possible that the population inhabiting the site was the mitmaq population resettled, within the confines of Inca labor system, from the other part of the Tawantinsuyu. In course of the archeological work, ten characteristic graves (chullpas) were discovered. These are rectangular buildings made of stone, up to two meters high, with a small entrance through which the bodies were placed inside [16,17]. Four chullpas (I – IV) were located in the center of the site and the other six in its close vicinity. The environmental conditions on the site were favorable for DNA preservation; the climate in the area is extremely dry, and the temperatures are low, often falling below zero. Moreover, the chullpas itself, protects bones from direct sunlight. 

### Samples

A total of 41 tooth or femur fragments were collected from six different chullpas from the Tompullo 2 site. The human remains had been disarticulated because of looting, and therefore to minimize the likelihood of taking two samples from the same individual, only teeth from the skull and pieces of left femurs were collected. To minimize post-excavation DNA degradation [30] most samples were collected directly from chullpas during field work in 2005. Face masks and gloves were worn during sampling to reduce the possibility of contamination of collected material. 14 additional samples excavated previously from the Tompullo 2 site were obtained from Museo Arqueológico de la Universidad Católica Santa María (Arequipa, Peru) (see: Additional file 1: Full list of samples from the Tompullo 2 archaeological site).

### Modern Amerindian sequences

MtDNA sequences of 715 individuals from 24 contemporary Amerindian populations from South America were obtained from the literature for comparison with ancient DNA sequences. Geographic localization of these populations and source of sequences are presented in Figure 1.

### Contamination precautions

DNA extraction and PCR setup were performed in a laboratory dedicated especially to ancient DNA work with positive air pressure. All staff wore lab coats, face masks and gloves. Working areas and benches were frequently cleaned with bleach and DNA ExitusPlus (Applichem). All reactions were carried out in laminar flow cabinets with HEPA filters. Only filter tips and sterile disposables were used. To eliminate the possibility of in-lab contamination negative controls were carried on with each DNA isolation procedure and PCR reaction. If PCR product was obtained in a negative control whole reaction was discarded and new aliquots of reagents were used in subsequent reactions. Additionally, mtDNA sequences and STR profiles were obtained for all the staff involved in the project.

### DNA extraction

Prior to DNA extraction, each tooth or bone fragment was submerged in bleach (6% w/v sodium hypochlorite) for 1 minute, cleaned with a sterile toothbrush and rinsed with ddH_2_O. Afterwards, samples were UV irradiated for at least 20 minutes on each side, and pulverized in a cryogenic mill (Spex CentriPrep). Up to 500 mg of bone or tooth powder was incubated overnight at 40°C in 1.6 ml of extraction buffer (0.5 M EDTA, 0.7 mg of proteinase K (20 mg/ml) (Bioline), 0.1 M DTT, 50 mM PTB, 0.5% N-Lauryl sarcosine) with constant agitation. Following incubation, the supernatant was extracted with phenol:chloroform:isoamyl alcohol (25:24:1, v:v:v), followed by two chloroform DNA extractions and isopropanol precipitation [31]. After precipitation, DNA was resuspended in 60 μl of ddH_2_O (Fluka). At least two independent DNA extractions were performed from each specimen.

### MtDNA amplification

For initial screening, a 146 bp amplicon of the mtDNA control region (mtCR) was amplified using the primer pair 16090 F (5'-ATT TCG TAC ATT ACT GCC AG-3') and 16236R (5^′^-GTG TGA TAG TTG AGG GTT GA-3^′^). If this product was successfully amplified, a longer (330 bp) fragment, encompassing positions 16111--16400 (HVRI), was amplified using the same forward primer 16090 F with a different reverse primer (16420R; 5^′^-TGA TTT CAC GGA GGA TGG TG-3^′^). Each reaction mix (25 μl) contained 0.2 μM of each primer, 2U of SAHARA Taq polymerase (Bioline), 0.1 M BSA, 10 mM dNTPs, 2.5 mM MgCl_2_ and 1.5 μl of extracted DNA. Cycling conditions consisted of an initial denaturation of 5 min. at 95°C followed by 40 cycles of 30 sec. at 95°C, 45 sec. at 56°C, 1 min. at 72°C followed by final extension of 7 min. at 72°C. PCR products were purified with SureClean Kit (Bioline) and sequenced using an ABI PRISM 3730 automated sequencer in DNA Sequencing & Oligonucleotide Synthesis Lab “Oligo.pl”. At least two PCR reactions were performed on each DNA sample. To ensure that the PCR products were homogenous and that the obtained sequences did not contain errors due to post-mortem DNA modification, each PCR product was cloned with the PCR Cloning Kit (Qiagen) according to the manufacturer’s instruction. One to ten clones were sequenced for each PCR product and the consensus sequence for each sample was generated with 50% majority rule (See: Additional file 2: Results of cloning of PCR products).

### Molecular sex determination

Each sample that yielded a reliable mtDNA sequence was also used for genetic sex determination. Fragments of the amelogenin and SRY genes were coamplified in a duplex PCR reaction with fluorescent labeled primers. Previously published primer sequences were used XYF: 5^′^ - Tamra – CCC TGG GCT CTG TAA AGA ATA GTG – 3^′^; XYR: 5^′^ – ATC AGA GCT TAA ACT GGG AAG CTG -3′ resulting in either a 106 bp product (female) or both 106 and 112 bp products (male). For SRYF: 5^′^ – Tamra - GCA CTT CGC TGC AGA GTA CCG A -3^′^ and SRYR: 5^′^ – ATA AGT ATC GAC CTC GTC GGA A – 3^′^ (resulting in a male specific 93 bp product) [32,33]. PCR reactions were carried out with the Multiplex PCR Kit (Qiagen), 25 μl of reaction mix contained 1x PCR Master Mix, 0.1 M BSA, 0.2 μM of XY primers, 0.1 μM SRY primers and 1.5 μl of DNA extract. Cycling conditions consisted of an initial denaturation of 15 min. at 95°C followed by 40 cycles of 30 sec. at 95°C, 90 sec. at 61°C, 45 sec. at 72°C followed by a final extension for 30 min. at 60°C. PCR products were electrophoresed in 3% agarose gels and analyzed on ABI PRISM 3730xl automated sequencer and Peak Scanner software (Applied Biosystems). At least two independent PCR reactions were performed for each DNA sample.

### Autosomal STR amplification

Twelve autosomal microsatellites (TH01, FGA, CSF1PO, D21S11, TPOX, D5S818, D8S1179, D16S539, vWA, D18S51, D13S317) were amplified in 3 multiplex reactions for each sample which had previously given reproducible mtDNA sequencing results. Primer sequences and final concentrations were in accordance with Butler and co-workers [34]. Reduced amplicon size multiplexes were specially designed for work with highly degraded DNA [34]. Moreover, extensive validation studies were undertaken for these sets [35,36]. PCR reactions were carried out in 25 μl of reaction mix containing 1x PCR Master Mix, 1.5 μl of DNA extract, 0.1 M BSA and appropriate concentrations of primers. Cycling conditions consisted of an initial denaturation of 15 min. at 95°C followed by 40 cycles of 30 sec. at 95°C, 90 sec. at 60°C, 45 sec. at 72°C followed by a final extension for 30 min. at 60°C. PCR products were analyzed on ABI PRISM 3730xl automated sequencer in Oligo.pl and Peak Scanner software (Applied Biosystems). For each locus, alleles were assigned according to allelic ladders from STR Base (http://www.cstl.nist.gov/div831/strbase). At least two independent PCR reactions were performed for each DNA sample. Alleles were scored when they appeared at least twice.

### Y-chromosome STR amplification

All the samples for which sex had been determined as male, were also typed for 16 Y-chromosome microsatellites (DYS456, DYS389I,II, DYS390, DYS458, DYS19, DYS385a,b, DYS393, DYS391, DYS439, DYS635, DYS392, H4, DYS437, DYS438 and DYS448) with AmpFℓSTR Kit (Applied Biosystems) according to the manufacturer’s recommendations with the exception that the number of PCR cycles was increased to 40. AmpFℓSTR Kit coamplifies 16 loci in one reaction and some of the amplicons are longer than 200 bp. As we encountered problems with amplification of longer amplicons with AmpFℓSTR Kit, all samples were additionally subjected to amplification of 15 Y-chromosome STRs with 3 “home made” multiplexes. Primers used in these multiplexes were designed to minimize the lengths of amplicons of each locus (see: Additional file 3: Details of Y chromosome STR multiplexes design and validation). At least two independent PCR reactions were performed from each DNA sample. Alleles were scored when they appeared at least twice.

### Statistical analysis

Pairwise genetic distances (F_ST_ based on mtDNA haplotype frequencies) between populations [37] were estimated with ARLEQUIN v. 3.0 software [38]. In this analysis, sequence length was cut down to 252 bp (16111--16362) to fit sequences from the literature. A matrix of pairwise distances was presented as an MDS plot constructed with SPSS software. For the microsatellite data, tests for Hardy-Weinberg equilibrium, H_O_, H_E_ and Na, were computed with ARLEQUIN v. 3.0 software.

Molecular data on mtDNA, autosomal and Y chromosomal genetic systems allow inference of kinship structure between individuals buried at Tompullo 2 site. MtDNA could provide evidence of maternal relationships. However HVRI haplotypes are considered as a weak marker for human identification and establishing kinship between individuals [39,40], as homoplasmy leads to identical haplotypes being carried by unrelated individuals. The “counting method” is usually employed to evaluate the significance of mtDNA HVRI match between the individuals [41,42]. The number of scores of desired haplotype in the database gives the probability that two haplotypes are identical by chance. Here we used a database with 715 sequences of modern Native Americans (Figure 1) to calculate the probability of identity of haplotype by chance.

To estimate pairwise relationships between each pair of individuals based on microsatellite data two relatedness coefficients were calculated. The Queller and Goodnight coefficient (R_QG_) [43] was calculated with Relatedness v. 5.0.8 software [44], while the Lynch and Ritland coefficient (R_LR_) [45] was calculated in GeneAlex v. 6.4 software [46]. Recent comparisons of the performance of relatedness coefficients suggest that use of Lynch and Ritland’s estimator results in the smallest sampling variances, but this estimator is sensitive for sample size and overall population relatedness [47]. Queller and Goodnight’s coefficient performs better when allele frequencies used in calculations are based on typing of a small and putatively related group of individuals which is the case for the samples from Tompullo 2 [48].

## Results

### Mitochondrial DNA

Of the 41 samples collected at Tompullo 2 site, we succeeded in amplification and sequencing of the 330 bp mtCR segment from 27 individuals. Amplification products in negative controls were obtained in three PCR reactions, these were discarded from further analyses. Two samples were rejected from further analysis because of inconsistencies in the sequences of the clones resulting from either DNA damage or in-lab contamination. One pair of samples (T2CH81 and T2CH82) showed identical mtDNA and autosomal STR genotypes. These samples were tooth and femur fragments collected from one grave and there is a high possibility that they belong to the same individual. Thus, one of these samples was excluded from further analysis.

**Figure 2:**
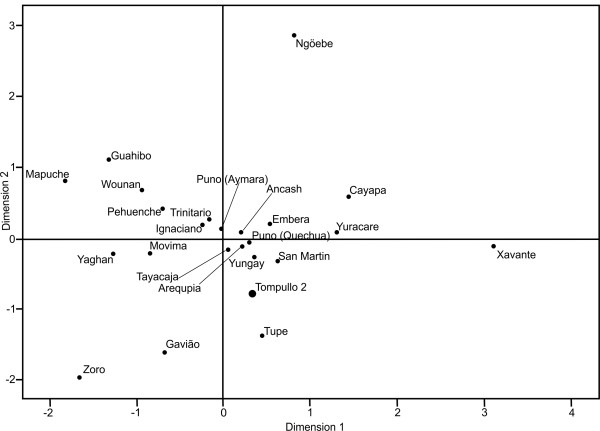
**Multidimensional Scaling plot based on pairwise distances (F**_**ST**_**) between Tompullo 2 and 23 contemporary populations.** The Ache population was very divergent from others and it was not used in multidimensional scaling analysis to retain figure legible.

Based on characteristic substitutions, each sequence was assigned to one of four major mtDNA haplogroups (Hgs) found in South America [49,50]. Out of 24 obtained sequences, 2 were assigned to haplogroup A2 (8.3%), 17 (70.8%) to haplogroup B2, 1 (4.2%) to C1 and 4 (16.7%) to D1. In sequences that belong to haplogroup B2, a diagnostic substitution T16189C resulted in the formation of a poly-C stretch (nt. 16184--16193), subsequently leading to poly-C length polymorphism in some sequences. This could have been a result of polymerase slippage during PCR or cloning [51], and thus poly-C length variation at this site was excluded from the analysis. Similarly, substitutions A16182C and A16183C were excluded because of possible dependence on the T16189C substitution [20,51]. HVRI haplotypes obtained in this study are presented in Table 1. Sequences were deposited in GenBank under accession numbers JN833681 - JN833704. Genetic distances between populations were estimated on the basis of pairwise F_ST_ (Figure 2, Additional file 4: Matrix of genetic distances (F_ST_) between Tompullo 2 and modern South American populations). Inhabitants of Tompullo 2 exhibit shorter distances to representatives of the Andean populations than to non-Andean ones, such as Peruvian populations from Puno, San Martin, Ancash, and Yungay. The shortest distances were observed between Tompullo 2 and Arequipa and Tayacaja populations (0.0294 and 0.0256, respectively). However, among Andean populations Fisher’s exact tests show significant differences in haplogroup frequencies between Tompullo2 and Tayacaja (*p* = 0.0273) as well as between Tompullo 2 and Puno (Quechua speaking) populations (*p* = 0.0427) .

**Table 1:** mtDNA HVRI (16111 – 16362) haplotypes obtained from Tompullo 2 inhabitants

**Sample**	**HVR-I (16111–16362)**	**Hg**
Tompullo 2		
	Chullpa I	
T2CH12	189 C, 217 C	B
T2CH13	223 T, 286 T, 325 C, 362 C	D
T2CH14	189 C, 217 C	B
T2CH16	168 T, 189 C, 217 C	B
	Chullpa III	
T2CH33	168 T, 189 C, 217 C	B
T2CH37	168 T, 189 C, 217 C	B
T2CH38	111 T, 217 C, 223 T, 290 T, 319A, 362 C	A
T2CH39	223 T, 286 T, 325 C, 362 C	D
	Chullpa VI	
T2CH61	168 T, 189 C, 217 C, 295 T	B
	Chullpa VII	
T2CH71	189 C, 223 T, 289 C, 310A, 325 C, 327 T	C
T2CH72	178 C, 189 C, 217 C	B
T2CH73	178 C, 189 C, 217 C	B
T2CH711	189 C, 217 C, 247 G, 261 T	B
T2CH712	189 C, 217 C, 247 G, 261 T	B
T2CH715	168 T, 189 C, 217 C, 295 T	B
T2CH719	223 T, 325 C, 362 C	D
T2CH728	178 C, 189 C, 217 C, 218 T	B
T2CH729	223 T, 325 C, 362 C	D
T2CH730	111 T, 217 C, 223 T, 290 T, 319A, 362 C	A
	Chullpa VIII	
T2CH82	189 C, 217 C, 289 G	B
T2CH83	168 T, 189 C, 217 C	B
T2CH84	168 T, 189 C, 217 C, 295 T	B
T2CH85	168 T, 189 C, 217 C, 295 T	B
T2CH86	168 T, 189 C, 217 C, 295 T	B

### Autosomal microsatellites

Each sample, for which mtDNA sequence was obtained, was typed for 12 autosomal microsatellites. Complete or nearly complete allelic profiles were obtained for 16 samples (Table 2). For each specimen, up to six PCR reactions from two different DNA extracts were performed in order to ensure the reliability of typing. Nevertheless, for locus D21S11, which has the longest amplicons, there were no amplification products in the case of three samples. Samples for which there were more than two loci of non-replicable typing were excluded from analysis. Expected (H_E_) and observed (H_O_) heterozygosity were 0.632 and 0.683, respectively (H_Mean_ = 0.656). The mean number of alleles per locus (N_A_) were 4.25. The mean heterozygosity obtained for the studied population falls within the range reported for Andean populations in a massive study by Wang and co-workers [52]. However, mean allele number (Na) is smaller than reported for contemporary and pre-Columbian South American populations (8.9 for Puna region, Argentina [53], 6.7 for Pampa Grande site, Salta, Argentina [54]). The population from Tompullo 2 did not display deviations from Hardy-Weinberg equilibrium. 

**Table 2:** Results of sex determination and genotypes of 12 autosomal microsatellites of 16 samples from Tompullo 2 site

**Sample**	**Sex**	**TH01**	**FGA**	**CSF1PO**	**D21S11**	**TPOX**	**D7S820**	**D5S818**	**D8S179**	**D16S539**	**vWA**	**D18S51**	**D13S317**
chullpa I													
T2CH12	F	7/7	24/26	13/13	32.2/32.2	13/13	12/13	11/11	13/15	10/11	17/17	16/16	10/13
T2CH13	M	7/10	20/28	11/13	30/32.2	9/9	11/11	7/11	13/16	13/13	17/17	13/18	10/10
T2CH14	M	7/7	20/24	12/13	31.2/32.2	12/13	11/12	10/11	13/15	10/13	17/17	16/16	10/13
T2CH16	M	7/7	20/25	12/13	29/32.2	12/13	11/13	7/11	15/16	10/13	16/17	19/19	10/13
chullpa II													
T2CH38	M	7/10	25/26	13/13	−/−	9/9	10/10	11/11	13/16	13/13	16/17	13/14	10/10
T2CH39	F	7/10	25/25	13/13	32.2/32.2	10/13	11/11	7/11	13/14	10/12	16/17	15/16	10/13
chullpa VII													
T2CH73	F	7/10	25/26	13/13	32.2/32.2	9/13	12/12	7/11	13/14	11/11	16/19	14/18	10/10
T2Ch715	M	7/10	25/26	13/13	32.2/32.2	9/13	12/12	7/7	−/−	10/11	17/19	13/14	10/12
T2CH719	M	7/10	20/26	12/13	29/32.2	9/13	11/12	7/11	13/15	11/14	17/17	14/16	10/13
T2CH729	M	7/10	20/20	12/13	−/−	9/13	12/12	7/11	13/15	11/14	17/17	14/16	10/13
T2CH730	M	7/10	20/26	12/13	30.2/32.2	9/9	11/12	7/11	15/15	11/11	17/17	13/19	10/11
chullpa VIII													
T2CH82	M	6/7	20/26	12/13	30.2/31	9/13	12/12	11/11	15/16	11/13	16/17	15/16	10/10
T2CH83	F	6/7	21/25	13/13	32.2/32.2	9/12	11/12	11/11	13/16	12/13	16/17	13/15	10/10
T2CH84	M	6/10	26/28	12/13	30/32.2	9/12	11/11	7/11	13/15	12/14	17/17	14/16	10/11
T2CH85	F	6/10	26/28	12/13	31.2/33.2	9/13	10/12	7/11	15/16	13/14	17/17	13/16	10/12
T2CH86	M	10/10	26/26	12/13	30/30	9/13	11/11	7/7	13/16	11/13	17/17	13/16	10/11

### Sex determination and Y chromosome microsatellites

Out of 25 samples tested for biological sex 16 yielded reproducible results (Table 2). 11 samples were determined as males and all were subsequently subjected to Y chromosome microsatellite typing (Table 3). The most complete profiles were obtained for individuals from chullpas I and VIII. In case of sample T2CH729 results were obtained only for five loci. Overall six distinct Y chromosome lineages were identified. Different Y chromosome lineages were present in each grave. In chullpa I, all three males carried the same genotype (alleles at 14 loci were obtained for all the three individuals), the same was in the case of chullpa VIII (at 11 loci). A different situation was observed in chullpa VII where three distinct lineages were present amongst four individuals.

**Table 3:** Genotypes of 16 Y chromosome microsatellites obtained for 11 individuals from Tompullo 2 site

**Individual**	**456**	**389I**	**390**	**389II**	**458**	**19**	**385a,b**	**393**	**391**	**439**	**635**	**392**	**H4**	**437**	**438**	**448**
Chullpa I																
T2CH13	15	14	23	30	16	13	15/19	14	10	14	21	16	12	14	11	20
T2CH14	15	14	23	30	16	13	15/19	14	10	14	-	-	12	14	11	20
T2CH16	15	14	23	30	16	13	15/19	14	10	14	21	-	12	14	11	20
Chullpa III																
T2CH38	15	14	-	-	16	16	15/19	-	-	11	-	16	-	15	-	18
Chullpa VII																
T2CH715	16	-	-	-	16	-	-	10	10	11	-	16	-	16	-	-
T2CH719	15	14	-	-	16	13	15/19	14	10	13	-	-	13	14	-	-
T2CH730	15	14	-	30	16	13	15/19	14	10	13	22	16	13	14	11	20
T2CH729	-	14	-	-	16	-	-	-	-	-	-	14	-	13	-	-
Chullpa VIII																
T2CH81	15	13	23	30	16	13	15/18	14	10	13	21	16	12	14	11	20
T2CH84	15	13	23	30	16	13	15/18	14	10	13	-	-	12	14	-	20
T2Ch86	15	13	23	-	16	13	15/18	14	10	13	21	16	12	14	-	20

### Genetic relationships between individuals

Eight distinct mtDNA haplotypes were shared between two or more individuals from the Tompullo 2 site (Table 4). The most common one (16168 T, 16189 C, 16217 C, 16295 T) was present in five individuals buried in three different chullpas. For haplotypes 16189 C, 16217 C and 16223 T, 16325 C, 16362 C which were carried by two pairs of individuals (T2CH12, T2CH14 and T2CH719, T2CH729, respectively) the probabilities of identity by chance were 0.094 and 0.057 high, respectively, but close relatedness between these pairs was confirmed by autosomal microsatellites analysis (see below). Haplotype 16168 T, 16189 C, 16217 C, carried by four individuals from Tompullo 2, was found 17 times in the database; the probability of encountering the identical allele in two individuals by chance equals 0.024. Only two out of the four individuals (T2CH16 and T2CH83) were successfully typed for autosomal microsatellites, and the calculated relatedness coefficients (R_QG__and LR_ = −0.01) does not confirm close relationship between those individuals. Thus based on available data, those individuals were not considered as maternal relatives. In all other cases, frequency of haplotype in the database varies between 0 (haplotypes 16189 C, 16217 C, 16247 G, 16261 T and 16223 T, 16286 T, 16325 C, 16362 C) and 0.001 (16168 T, 16189 C, 16217 C, 16295 T) and the individuals carrying these haplotypes were considered to be maternal relatives (Table 4).

**Table 4:** Individuals with identical mtDNA haplotypes

**Individuals**	**Chullpa**	**Haplotype**	**In database**	**Frequency**	**95% CI**
T2CH12	I	189 C, 217 C	67	0.094	0.072-0.115
T2CH14	I				
T2CH719	VII	223 T, 325 C, 362 C	40	0.056	0.039-0.073
T2CH729	VII				
T2CH72	VII	178 C, 189 C, 217 C	1	0.001	0-0.004
T2CH73	VII				
T2CH16	I	168 T, 189 C, 217 C	17	0.024	0.013-0.035
T2CH33	III				
T2CH37	III				
T2CH83	VIII				
T2CH38	III	111 T, 217 C, 223 T, 290 T, 319A, 362 C	1	0.001	0-0.004
T2CH730	VII				
T2CH61	VI	168 T, 189 C, 217 C, 295 T	1	0.001	0-0.004
T2CH715	VII				
T2CH84	VIII				
T2CH85	VIII				
T2CH86	VIII				
T2CH711	VII	189 C, 217 C, 247 G, 261 T	0	0	0-0.004
T2CH712	VII				
T2CH13	I	223 T, 286 T, 325 C, 362 C	0	0	0-0.004
T2CH39	III				

Number of the same haplotypes in database of 715 sequences, frequency of each haplotype and 95% confidence intervals.

Due to the uniparental mode of inheritance of mtDNA, all maternal relatives possess identical haplotype and it is impossible to infer the degree of relationship between individuals. We estimated the degree of relatedness between these individuals by calculating R coefficients based on microsatellite data. Using both estimators, related pairs of individuals were found buried in one chullpa (e.g. individuals T2CH12 and T2CH14 that share the same mtDNA haplotype and have R_QG_ = 0.54 and R_LR_ = 0.19 or T2CH84 and T2CH86 with the same mtDNA haplotype and R_QG_ = 0.39 and R_LR_ = 0.15), as well as buried in different chullpas (e.g. individuals T2CH13 and T2CH86 with different mtDNA haplotypes and R_QG_ = 0.39 and R_LR_ = 0.1 or T2CH38 and T2CH83 with R_QG_ = 0.43 and R_LR_ = 0.07). See Additional file 5: Matrix of relatedness coefficients (R_QG_ and R_LR_) estimated for individuals from Tompullo 2 site, for all R coefficients.

For both estimators the mean relatedness calculated for all pairs of individuals buried in the same grave (R_QG_ = 0.082 and R_LR_ = 0.024) was significantly (two tailed t-test, p = 0.0116 and p = 0.0049) higher than for all pairs of individuals buried in different graves (R_QG_ = −0.073 and R_LR_ = − 0.05).

## Discussion

Appropriate verification of results is crucial in all DNA studies of human remains [55-58], as modern DNA contamination and post-mortem DNA damage can lead to biased results and misleading conclusions [59]. In this study strict precautions were taken to avoid the contamination of samples with modern DNA during sampling and laboratory analysis, with appropriate effort to exclude possible errors in the obtained sequences. The skeletal material from the Tompullo 2 site was of extraordinary macroscopic preservation and the environmental conditions at the site strongly favored DNA preservation. Data from autosomal and Y chromosome microsatellite typing also strongly support the authenticity of the results as no triple peaks were observed throughout the analysis. Identical profiles obtained for samples T2CH81 and T2CH82 were most probably an effect of sampling both teeth and femur from one individual. Mean heterozygosity is comparable to that reported for modern Amerindian populations, which indicates that no large allele drop outs occurred during microsatellite typing.

Mitochondrial DNA analysis points to the Andean origin of the studied population. Inhabitants of Tompullo 2 exhibit a high frequency of haplogroup B2, characteristic for most contemporary and pre-Columbian Andean populations [23,25,60]. Based on genetic distance (F_ST_), Tompullo 2 residents were most similar to contemporary Andean populations, especially those from the same geographic area. Samples from contemporary populations were collected from Quechua or Aymara speakers from Peruvian cities (Puno, Yungay, San Martin) [23,25] and from farming communities, which settled in the region before the arrival of Europeans (Arequipa, Tayacaja, Ancash) [24,61]. Despite differences in haplogroup frequencies between Tompullo 2, Tayacaja and Quechua speakers from Puno, our data suggest continuity of populations inhabiting the region for at least the last five hundred years, without major change in mtDNA diversity. This confirms that the impact of European colonization on Native Amerindian populations was relatively small in the Andean region, and is congruent with historical data [62].

Kinship analyses using both uniparentally (mtDNA, Y-STRs) and biparentally (autosomal STRs) inherited markers indicate a complex pattern of relationships between individuals. On the one hand mtDNA and autosomal microsatellite analyses indicate closely related individuals buried in the same, as well as in different, chullpas. However, mean relatedness coefficients between individuals buried in the same chullpa were significantly higher than those between pairs of individuals buried in different chullpas, suggesting that degree of relationship was one of the factors influencing mortuary practices. Moreover, Y chromosome microsatellites revealed that in at least two graves (chullpa I and III), all male individuals were kindred, possessing identical Y chromosome profiles. Relatedness coefficients indicate that those males were rather distant relatives and probably belonged to different generations. These data suggest that the community from Tompullo 2 site was composed of patrilineal family groups and members of each family group were buried in one grave.

Theoretical considerations of Andean kinship are most often based on the Juan Pérez Bocanegra diagram of Inca Kinship [63]. Implications from this diagram on ayllu organization were depicted in details by Isbell [11]. The model assumes that a couple that will marry should not belong to one ayllu. The groom’s family should offer his sister to marry the bride’s brother (so-called sister exchange). In the Andean communities, kin relation was recognized to the fourth level of kinship (third cousins) and marriages between relatives closer than third cousins were prohibited. These conditions result in a kinship scheme (Fig. 7.19 in [11]) that involves marriages between four distinct groups (families) in four generation cycles. Identification of such model from archaeological record is usually impossible; however, Isbell [11] describes chullpas from Chota-Cutervo region that show architectonic features and internal organization reflecting the ideal ayllu model.

In the case of Tompullo 2, detailed reconstruction of genealogical trees of each family or group was impossible due to the small sample size, but we believe that the observed pattern of burial could be a result of social organization similar to that presented by Isbell. Members of each group were buried in distinct chullpas that served as family graves, while the relationship shown between individuals found in different chullpas were an effect of marriages between members of distinct groups, resulting in the inclusion of the bride into the grooms family and finally in her burial in the husband’s family grave.

This scenario is, however, altered by the results from chullpa VII where, from 4 male individuals, 3 different Y chromosome lineages were found. Individuals T2CH719 and T2CH730 possess an identical Y–STR profile. Individual T2CH729 carries a different Y chromosome, but has the same mtDNA as individual T2CH719 and R coefficients (R_QG_ = 0.76 and R_LR_ = 0.43) indicates that they were close relatives. Most probably they were sons of one mother and different fathers. This is not a case of individual T2CH715, autosomal microsatellites analysis indicates its close relationship to individual T2CH73 (female) buried in the same chullpa (R_QG_ = 0.64 and R_LR_ = 0.26). They yielded different mtDNA haplotype and thus the only possible relationship explaining their high relatedness coefficient is that they share one father and two different mothers.

There is no evidence that chullpa VII somehow differs architecturally or archaeologically from the other chullpas found at Tompullo 2 [16]. Thus, there is no proof that this grave was used by a group any different from other inhabitants of this site. However, as it is stated by Isbell [11], the rules governing marriages and social organization were an idealization, and we cannot exclude a situation that was intentionally or unintentionally violated in some situations.

## Conclusions

Genetic analyses of kinship relationships between individuals buried on the Tompullo 2 archaeological site reveal that individuals buried in the same chullpa were more closely related than those buried in different chullpas; moreover, all males buried in one chullpa share identical Y chromosome profiles (except for chullpa VII). These leads to the conclusion that this community was organized into patrilineal family groups. The use of chullpas as family graves is consistent with the idea of ayllu-based communities with kinship relationships as a foundation for each group. However, open sepulchers or chullpas were widely distributed in the Central Andes during the Late Horizon and many different types of such mortuary constructions could be found on sites with different ethnoarchaeological backgrounds [11]. Thus we cannot be sure that the model of social organization inferred in the Tompullo 2 site was universal for all Andean communities.

## Authors’ contribution

MB, AS, MS designed the study and collected the samples. MB performed the experiments and analyzed the data. KD developed and validated Y chromosome multiplexes. MB, MS and PW wrote the manuscript. All authors read and approved the final manuscript.

## Supplementary Material

Additional file 1 Full list of samples from Tompullo 2 archaeological site. Click here for file

Additional file 2 Results of cloning of PCR products. Click here for file

Additional file 3 Details of Y chromosome STR multiplexes design and validation. Click here for file

Additional file 4 **Matrix of genetic distances (F**_**ST**_**) between Tompullo 2 and modern South American populations.** Click here for file

Additional file 5 ** Matrix of relatedness coefficients (R**_**QG**_**and R**_**LR**_**) estimated for individuals from Tompullo 2 site.** Click here for file
