# Supplementary material for: Ancient DNA reveals kinship burial patterns of a pre-Columbian Andean community
Source: BMC Genet. 2012 Apr 23;13:30. doi: 10.1186/1471-2156-13-30 (PMC3470988; doi:10.1186/1471-2156-13-30)
Supplement: Additional file 2 — Results of cloning of PCR products. [file 1471-2156-13-30-S2.doc]

**Additional file 2**: Results of cloning of PCR products.

Sample T2CH12

16120 16130 16140 16150 16160 16170 16180 16190 16200 16210

....|....|....|....|....|....|....|....|....|....|....|....|....|....|....|....|....|....|....|....|

CRS CCACCATGAATATTGTACGGTACCATAAATACTTGACCACCTGTAGTACATAAAAACCCAATCCACATCAAAACCCCCTCCCCATGCTTACAAGCAAGTA

T2CH12_PCRI ------------............................................................C.....C.....................

T2CH12_PCRII -----------------.......................................................C.....C.....................

T2CH12_I_8 ----------------------------------------------------------------........C.....C.....................

T2CH12_I_11 ------------------------------------------------------------------------......C.....................

T2CH12_I_3 --------------------------------------------------------................C.....C.....................

T2CH12_I_1 ........................................................................C.....C.....................

T2CH12_I_5 ........................................................................C.....C.....................

T2CH12_I_7 ........................................................................C.....C.....................

T2CH12_I_9 .................................C......................................C.....C.....................

T2CH12_I_2 ........................................................................C...--C.....................

T2CH12_II_2 ........................................................................-.....C...-.................

T2CH12_II_1 ----------------------------------------------------------------------------------------------------

T2CH12_II_3 ----------------------------------------------------------------------------------------------------

16220 16230 16240 16250 16260 16270 16280 16290 16300 16310

....|....|....|....|....|....|....|....|....|....|....|....|....|....|....|....|....|....|....|....|

CRS CAGCAATCAACCCTCAACTATCACACATCAACTGCAACTCCAAAGCCACCCCTCACCCACTAGGATACCAACAAACCTACCCACCCTTAACAGTACATAG

T2CH12_PCRI ......C.............................................................................................

T2CH12_PCRII ......C.............................................................................................

T2CH12_I_8 ......C...T.........................................................................................

T2CH12_I_11 ......C.............................................................................................

T2CH12_I_3 ......C..G..........................................................................................

T2CH12_I_1 ......C...............................................................................C.............

T2CH12_I_5 ......C..............................................T..............................................

T2CH12_I_7 ......C.............................................................................................

T2CH12_I_9 ......C.......A................................................................................T....

T2CH12_I_2 ......C.............................................................................................

T2CH12_II_2 ......C.............................................................................................

T2CH12_II_1 ------C.............................................................................................

T2CH12_II_3 -----.C.............................................................................................

Sample T2CH12 - continued

16320 16330 16340 16350 16360 16370 16380 16390 16400

....|....|....|....|....|....|....|....|....|....|....|....|....|....|....|....|....|....|

CRS TACATAAAGCCATTTACCGTACATAGCACATTACAGTCAAATCCCTTCTCGTCCCCATGGATGACCCCCCTCAGATAGGGGTCCCTTGAC

T2CH12_PCRI ...................................................................-----------------------

T2CH12_PCRII ..............................................................----------------------------

T2CH12_I_8 ..........................................................................................

T2CH12_I_11 ..........................................................................................

T2CH12_I_3 ..........................................................................................

T2CH12_I_1 ..........................................................................................

T2CH12_I_5 ..........................................................................................

T2CH12_I_7 ..........................................................................................

T2CH12_I_9 ..........................................................................................

T2CH12_I_2 ..........................................................................................

T2CH12_II_2 ..........................................................................................

T2CH12_II_1 ..........................................................................................

T2CH12_II_3 ..........................................................................................

Sample T2CH13

16120 16130 16140 16150 16160 16170 16180 16190 16200 16210

....|....|....|....|....|....|....|....|....|....|....|....|....|....|....|....|....|....|....|....|

CRS CCACCATGAATATTGTACGGTACCATAAATACTTGACCACCTGTAGTACATAAAAACCCAATCCACATCAAAACCCCCTCCCCATGCTTACAAGCAAGTA

T2CH13_PCRI ....................................................................................................

T2CH13_PCRII ....................................................................................................

T2CH13_I_2 ....................................................................................................

T2CH13_I_3 ....................................................................................................

T2CH13_I_4 ....................................................................................................

T2CH13_I_5 ....................................................................................................

T2CH13_I_6 ...................................C................................................................

T2CH13_I_7 ....................................................................................................

T2CH13_I_8 ....................................................................................................

T2CH13_I_13 ....................................................................................................

T2CH13_I_17 ....................................................................................................

T2CH13_II_11 ......................................................G.............................................

T2CH13_II_14 ....................................................................................................

T2CH13_III_1 ....................................................................................................

16220 16230 16240 16250 16260 16270 16280 16290 16300 16310

....|....|....|....|....|....|....|....|....|....|....|....|....|....|....|....|....|....|....|....|

CRS CAGCAATCAACCCTCAACTATCACACATCAACTGCAACTCCAAAGCCACCCCTCACCCACTAGGATACCAACAAACCTACCCACCCTTAACAGTACATAG

T2CH13_PCRI ............T..............................................................T........................

T2CH13_PCRII ............T..............................................................T........................

T2CH13_I_2 ............T..............................................................T........................

T2CH13_I_3 ............T..............................................................T........................

T2CH13_I_4 ............T..............................................................T........................

T2CH13_I_5 ............T..............................................................T........................

T2CH13_I_6 ............T..............................................................T........................

T2CH13_I_7 ............T..............................................................T........................

T2CH13_I_8 ............T...............................................C..............T........................

T2CH13_I_13 ............T..............................................................T........................

T2CH13_I_17 ............T.T.........................................T..................T........................

T2CH13_II_11 ............T..............................................................T........................

T2CH13_II_14 ............T..............................................................T........................

T2CH13_III_1 ............T..............................................................T........................

Sample T2CH13 - continued

16320 16330 16340 16350 16360 16370 16380 16390 16400

....|....|....|....|....|....|....|....|....|....|....|....|....|....|....|....|....|....|

CRS TACATAAAGCCATTTACCGTACATAGCACATTACAGTCAAATCCCTTCTCGTCCCCATGGATGACCCCCCTCAGATAGGGGTCCCTTGAC

T2CH13_PCRI ..............C....................................C......................................

T2CH13_PCRII ..............C....................................C......................................

T2CH13_I_2 ..............C....................................C............................A.........

T2CH13_I_3 ..............C....................................C......................................

T2CH13_I_4 ..............C....................................C......................................

T2CH13_I_5 ..............C....................................C......................................

T2CH13_I_6 ..............C....................................C......................................

T2CH13_I_7 ..............C...................G................C......................................

T2CH13_I_8 ..............C....................................C......................................

T2CH13_I_13 ..............C....................................C......................................

T2CH13_I_17 ..............C....................................C......................................

T2CH13_II_11 ..............C....................................C......................................

T2CH13_II_14 ..............C....................................C......................................

T2CH13_III_1 ..............C....................................C......................................

Sample T2CH14

16120 16130 16140 16150 16160 16170 16180 16190 16200 16210

....|....|....|....|....|....|....|....|....|....|....|....|....|....|....|....|....|....|....|....|

CRS CCACCATGAATATTGTACGGTACCATAAATACTTGACCACCTGTAGTACATAAAAACCCAATCCACATCAAAACCCCCTCCCCATGCTTACAAGCAAGTA

T2CH14_PCRI ------------............................................................C.....C.....................

T2CH14_PCRII ------------............................................................C.....C.....................

T2CH14_I_1 ........................................................................C.....C...-.............G...

T2CH14_I_2 ........................................................................C.....C...------------------

T2CH14_I_3 ........................................................................C.....C.....................

T2CH14_II_2 ........................................................................C.....C.....................

T2CH14_II_3 ..............................................................T.........C.....C.....................

T2CH14_II_1 ........................................................................-.....C.....................

T2CH14_III_1 ---------------------------------------------------.....................C.....C.....................

16220 16230 16240 16250 16260 16270 16280 16290 16300 16310

....|....|....|....|....|....|....|....|....|....|....|....|....|....|....|....|....|....|....|....|

CRS CAGCAATCAACCCTCAACTATCACACATCAACTGCAACTCCAAAGCCACCCCTCACCCACTAGGATACCAACAAACCTACCCACCCTTAACAGTACATAG

T2CH14_PCRI ......C.............................................................................................

T2CH14_PCRII ......C.............................................................................................

T2CH14_I_1 ......C..................................................................................G..........

T2CH14_I_2 ----------------------------------------------------------------------------------------------------

T2CH14_I_3 ..........................--------------------------------------------------------------------------

T2CH14_II_2 ......C.............................................................................................

T2CH14_II_3 ......C.............................................................................................

T2CH14_II_1 ......C............................................................T................................

T2CH14_III_1 ......C.............................................................................................

16320 16330 16340 16350 16360 16370 16380 16390 16400

....|....|....|....|....|....|....|....|....|....|....|....|....|....|....|....|....|....|

CRS TACATAAAGCCATTTACCGTACATAGCACATTACAGTCAAATCCCTTCTCGTCCCCATGGATGACCCCCCTCAGATAGGGGTCCCTTGAC

T2CH14_PCRI ...............................................................................-----------

T2CH14_PCRII ...........................................................................---------------

T2CH14_I_1 ..........................................................A...............................

T2CH14_I_2 ------------------------------------------------------------------------------------------

T2CH14_I_3 ------------------------------------------------------------------------------------------

T2CH14_II_2 ............................T.............................................................

T2CH14_II_3 .......G.........................T........................................................

T2CH14_II_1 C.........................................................................................

T2CH14_III_1 ..........................................................................................

Sample T2CH16

16120 16130 16140 16150 16160 16170 16180 16190 16200 16210

....|....|....|....|....|....|....|....|....|....|....|....|....|....|....|....|....|....|....|....|

CRS CCACCATGAATATTGTACGGTACCATAAATACTTGACCACCTGTAGTACATAAAAACCCAATCCACATCAAAACCCCCTCCCCATGCTTACAAGCAAGTA

T2CH16_PCRI ----------...............................................T..............C.....C.....................

T2CH16_PCRII ------------.............................................T..............C.....C.....................

T2CH16_I_1 .........................................................T..............C.....C.....................

T2CH16_I_3 .........................................................T..............C.....C.....................

T2CH16_II_1 .........................................................T..............C.....C.....................

T2CH16_II_2 .........................................................T..............C.....C......---------------

T2CH16_II_3 .........................................................T..............C.....C...-.................

16220 16230 16240 16250 16260 16270 16280 16290 16300 16310

....|....|....|....|....|....|....|....|....|....|....|....|....|....|....|....|....|....|....|....|

CRS CAGCAATCAACCCTCAACTATCACACATCAACTGCAACTCCAAAGCCACCCCTCACCCACTAGGATACCAACAAACCTACCCACCCTTAACAGTACATAG

T2CH16_PCRI ......C.............................................................................................

T2CH16_PCRII ......C.............................................................................................

T2CH16_I_1 ......C.............................................................................................

T2CH16_I_3 ......C.............................................................................................

T2CH16_II_1 ......C.............................................................................................

T2CH16_II_2 ----------------------------------------------------------------------------------------------------

T2CH16_II_3 ......C.............................................................................................

16320 16330 16340 16350 16360 16370 16380 16390 16400

....|....|....|....|....|....|....|....|....|....|....|....|....|....|....|....|....|....|

CRS TACATAAAGCCATTTACCGTACATAGCACATTACAGTCAAATCCCTTCTCGTCCCCATGGATGACCCCCCTCAGATAGGGGTCCCTTGAC

T2CH16_PCRI ...............................................................................A..--------

T2CH16_PCRII ...............................................................................A....------

T2CH16_I_1 ...............................................................................A..........

T2CH16_I_3 ...............................................................................A..........

T2CH16_II_1 ...............................................................................A..........

T2CH16_II_2 ------------------------------------------------------------------------------------------

T2CH16_II_3 ...............................................................................A..........

Sample T2CH33

16120 16130 16140 16150 16160 16170 16180 16190 16200 16210

....|....|....|....|....|....|....|....|....|....|....|....|....|....|....|....|....|....|....|....|

CRS CCACCATGAATATTGTACGGTACCATAAATACTTGACCACCTGTAGTACATAAAAACCCAATCCACATCAAAACCCCCTCCCCATGCTTACAAGCAAGTA

T2CH33_PCRI ---------................................................T..............C.....C.....................

T2CH33_PCRII -----------..............................................T..............C.....C.....................

T2CH33_I_2 .........................................................T....T.........C.....C..............A......

T2CH33_I_3 ......................TT.................................T..............C.....C.....................

T2CH33_I_4 .................T..............C........................T..............C.....C...-.................

T2CH33_I_6 ---------------------------------------------------------------------...C.....C.....................

T2CH33_I_7 ---------------------------------------------------------T..............C.....C...-..............A..

T2CH33_I_1 ......................TT.................................T..............C.....C.....................

T2CH33_I_10 .........................................................T..............C.....C.....................

T2CH33_I_11 .........................................................T..............C.....C...-.................

T2CH33_I_12 .........................................................T..............C.....C.....................

T2CH33_II_1 ................................C........................T..............C.....C.......T.......T.....

T2CH33_II_2 ...........G...........T.................................T..............C.....C.....................

16220 16230 16240 16250 16260 16270 16280 16290 16300 16310

....|....|....|....|....|....|....|....|....|....|....|....|....|....|....|....|....|....|....|....|

CRS CAGCAATCAACCCTCAACTATCACACATCAACTGCAACTCCAAAGCCACCCCTCACCCACTAGGATACCAACAAACCTACCCACCCTTAACAGTACATAG

T2CH33_PCRI ......C.................................Y..................Y........................................

T2CH33_PCRII ......C.............................................................................................

T2CH33_I_2 ......C.............................................................................................

T2CH33_I_3 .....................................T............T......T.T................T.......................

T2CH33_I_4 ......C....................................................T........................................

T2CH33_I_6 ......C.................................T...........................................................

T2CH33_I_7 ..A...C.............................................................................................

T2CH33_I_1 .....................................T............T......T.T................T.......................

T2CH33_I_10 ......C.................................T...........................................................

T2CH33_I_11 ......C.................................T...........................................................

T2CH33_I_12 ......C.................................T...........................................................

T2CH33_II_1 ......C.............................................................................................

T2CH33_II_2 ......C.............................................................................................

Sample T2CH33 – continued

16320 16330 16340 16350 16360 16370 16380 16390 16400

....|....|....|....|....|....|....|....|....|....|....|....|....|....|....|....|....|....|

CRS TACATAAAGCCATTTACCGTACATAGCACATTACAGTCAAATCCCTTCTCGTCCCCATGGATGACCCCCCTCAGATAGGGGTCCCTTGAC

T2CH33_PCRI ..............................................................................------------

T2CH33_PCRII .............................................................................-------------

T2CH33_I_2 ...............................................................................A..........

T2CH33_I_3 ...............................................................................A..........

T2CH33_I_4 .................................................T.............................A..........

T2CH33_I_6 ...............................................................................A..........

T2CH33_I_7 ...............................................................................A..........

T2CH33_I_1 ...............................................................................A..........

T2CH33_I_10 ...............................................................................A..........

T2CH33_I_11 ...............................................................................A..........

T2CH33_I_12 ...............................................................................A..........

T2CH33_II_1 ...............................................................................A.........T

T2CH33_II_2 ................................................................T..............A..........

Sample T2CH37

16120 16130 16140 16150 16160 16170 16180 16190 16200 16210

....|....|....|....|....|....|....|....|....|....|....|....|....|....|....|....|....|....|....|....|

CRS CCACCATGAATATTGTACGGTACCATAAATACTTGACCACCTGTAGTACATAAAAACCCAATCCACATCAAAACCCCCTCCCCATGCTTACAAGCAAGTA

T2CH37_PCRII -----------..............................................T..............C.....C.....................

T2CH37_PCRI -----------....-.........................................t..............C.....C.....................

T2CH37_I_1 --------------------.....................................T..............C.....C.....................

T2CH37_I_2 .........................................................T..............C.....C...------------------

T2CH37_I_1_SH ------------------------------------------------------------------------------------------..........

T2CH37_I_2_SH ------------------------------------------------------------------------------------------..........

T2CH37_I_3_SH ------------------------------------------------------------------------------------------..........

T2CH37_I_4_SH ------------------------------------------------------------------------------------------..........

T2CH37_I_5_SH ------------------------------------------------------------------------------------------..........

T2CH37_I_1_SH1 ------------------------------------------------------------------------------------------..........

T2CH37_I_2_SH1 ------------------------------------------------------------------------------------------..........

T2CH37_I_3_SH1 ------------------------------------------------------------------------------------------..........

T2CH37_II_1 .........................................................T..............C.....C.................T...

T2CH37_II_2 .........................................................T..............C.....C.....................

T2CH37_II_3 ---------------------------------------------------------------------...C.....C.....................

T2CH37_III_1 .........................................................T..............C.....C.....................

16220 16230 16240 16250 16260 16270 16280 16290 16300 16310

....|....|....|....|....|....|....|....|....|....|....|....|....|....|....|....|....|....|....|....|

CRS CAGCAATCAACCCTCAACTATCACACATCAACTGCAACTCCAAAGCCACCCCTCACCCACTAGGATACCAACAAACCTACCCACCCTTAACAGTACATAG

T2CH37_PCRII ......C.............................................................................................

T2CH37_PCRI ......C.............................................................................................

T2CH37_I_1 ......C.............................................................................................

T2CH37_I_2 ----------------------------------------------------------------------------------------------------

T2CH37_I_1_SH ......C.........................................................................................----

T2CH37_I_2_SH ......C.........................................................................................----

T2CH37_I_3_SH ......C.........................................................................................----

T2CH37_I_4_SH ......C.........................................................................................----

T2CH37_I_5_SH ......C.........................................................................................----

T2CH37_I_1_SH1 ......C.............................................................................................

T2CH37_I_2_SH1 ......C.............................................................................................

T2CH37_I_3_SH1 ......C.............................................................................................

T2CH37_II_1 ......C.......................TG............C..C..............A...................C.................

T2CH37_II_2 ...T..C.............................................................................................

T2CH37_II_3 ......C.............................................................................................

T2CH37_III_1 ......C.............................................................................................

Sample T2CH37 – continued

16320 16330 16340 16350 16360 16370 16380 16390 16400

....|....|....|....|....|....|....|....|....|....|....|....|....|....|....|....|....|....|

CRS TACATAAAGCCATTTACCGTACATAGCACATTACAGTCAAATCCCTTCTCGTCCCCATGGATGACCCCCCTCAGATAGGGGTCCCTTGAC

T2CH37_PCRII .............................................................................-------------

T2CH37_PCRI ...............................................................................-----------

T2CH37_I_1 ................................................................--------------------------

T2CH37_I_2 ------------------------------------------------------------------------------------------

T2CH37_I_1_SH ------------------------------------------------------------------------------------------

T2CH37_I_2_SH ------------------------------------------------------------------------------------------

T2CH37_I_3_SH ------------------------------------------------------------------------------------------

T2CH37_I_4_SH ------------------------------------------------------------------------------------------

T2CH37_I_5_SH ------------------------------------------------------------------------------------------

T2CH37_I_1_SH1 ...............................................................................A..........

T2CH37_I_2_SH1 ...............................................................................A..........

T2CH37_I_3_SH1 ...............................................................................A..........

T2CH37_II_1 ...........T............................................G....A.................A..........

T2CH37_II_2 .............................................C.................................A..........

T2CH37_II_3 ...............................................................................A..........

T2CH37_III_1 ......................................-.......................----------------------------

Sample T2CH38

PCR product from second DNA isolation (II) was cloned without direct sequencing, thus only sequences of clones are reported.

16120 16130 16140 16150 16160 16170 16180 16190 16200 16210

....|....|....|....|....|....|....|....|....|....|....|....|....|....|....|....|....|....|....|....|

CRS CCACCATGAATATTGTACGGTACCATAAATACTTGACCACCTGTAGTACATAAAAACCCAATCCACATCAAAACCCCCTCCCCATGCTTACAAGCAAGTA

T2CH38_PCRI T...................................................................................................

T2CH38_I_1 -------.........G...................................................................................

T2CH38_I_2 T...................................................................................................

T2CH38_II_1 T.G.............G...................................................................................

T2CH38_II_2 T...................................................................................................

T2CH38_II_4 T...................................................................................................

T2CH38_II_5 T..........................................................................................G........

T2CH38_II_11 T...................................................................................................

16220 16230 16240 16250 16260 16270 16280 16290 16300 16310

....|....|....|....|....|....|....|....|....|....|....|....|....|....|....|....|....|....|....|....|

CRS CAGCAATCAACCCTCAACTATCACACATCAACTGCAACTCCAAAGCCACCCCTCACCCACTAGGATACCAACAAACCTACCCACCCTTAACAGTACATAG

T2CH38_PCRI ......C.....T..............................................................Y...T....................

T2CH38_I_1 ......C.....T..................................................................T....................

T2CH38_I_2 ......C.....T..................................................................T....................

T2CH38_II_1 ......C.....T..................................................................T....................

T2CH38_II_2 ......C.....T..................................................................T....................

T2CH38_II_4 ......C.....T..................................................................T....................

T2CH38_II_5 ......C.....T..................................................................T....................

T2CH38_II_11 ......C.....T..................................................................T....................

Sample T2CH38 – continued

16320 16330 16340 16350 16360 16370 16380 16390 16400

....|....|....|....|....|....|....|....|....|....|....|....|....|....|....|....|....|....|

CRS TACATAAAGCCATTTACCGTACATAGCACATTACAGTCAAATCCCTTCTCGTCCCCATGGATGACCCCCCTCAGATAGGGGTCCCTTGAC

T2CH38_PCRI ........A..........................................C......................................

T2CH38_I_1 ........A..................................T.......C......................................

T2CH38_I_2 ........A..........................................C......................................

T2CH38_II_1 ........A..................................T.......C......................................

T2CH38_II_2 ........A..........................................C......................................

T2CH38_II_4 ........A..........................................C......................................

T2CH38_II_5 ........A.............G............................C......................................

T2CH38_II_11 ........A..........................................C......................................

Sample T2CH39

16120 16130 16140 16150 16160 16170 16180 16190 16200 16210

....|....|....|....|....|....|....|....|....|....|....|....|....|....|....|....|....|....|....|....|

CRS CCACCATGAATATTGTACGGTACCATAAATACTTGACCACCTGTAGTACATAAAAACCCAATCCACATCAAAACCCCCTCCCCATGCTTACAAGCAAGTA

T2CH39_PCRI ....................................................................................................

T2CH39_PCRII ....................................................................................................

T2CH39_I_1 ............C.......................................................................................

T2CH39_I_2 ....................................................................................................

T2CH39_I_3 ....................................................................................................

T2CH39_I_5 ....................................................................................................

T2CH39_I_6 ................G...................................................................................

T2CH39_I_7 ....................................................................................................

T2CH39_I_8 ....................................................................................................

T2CH39_I_9 ....................................................................................................

T2CH39_I_10 ....................................................................................................

T2CH39_II_1 -------------------------.............-.....G.......................................................

T2CH39_II_2 ....................................................................................................

T2CH39_III_2 ....................................................................................................

T2CH39_III_2 .....................................................................G..............................

16220 16230 16240 16250 16260 16270 16280 16290 16300 16310

....|....|....|....|....|....|....|....|....|....|....|....|....|....|....|....|....|....|....|....|

CRS CAGCAATCAACCCTCAACTATCACACATCAACTGCAACTCCAAAGCCACCCCTCACCCACTAGGATACCAACAAACCTACCCACCCTTAACAGTACATAG

T2CH39_PCRI ............T..............................................................T........................

T2CH39_PCRII ............T..............................................................T........................

T2CH39_I_1 ............T..............................................................T........................

T2CH39_I_2 ....................................................................................................

T2CH39_I_3 ............T..............................................................T........................

T2CH39_I_5 ............T..............................................................T........................

T2CH39_I_6 ............T..............................................................T........................

T2CH39_I_7 ............T..............................................................T........................

T2CH39_I_8 ............T..............................................................T........................

T2CH39_I_9 ............T..............................................................T........................

T2CH39_I_10 ............T..............................................................T........................

T2CH39_II_1 ............T..............................................................T........................

T2CH39_II_2 ............T..............................................................T........................

T2CH39_III_2 ............T..............................................................T........................

T2CH39_III_2 ............T..............................................................T........................

Sample T2CH39 – continued

16320 16330 16340 16350 16360 16370 16380 16390 16400

....|....|....|....|....|....|....|....|....|....|....|....|....|....|....|....|....|....|

CRS TACATAAAGCCATTTACCGTACATAGCACATTACAGTCAAATCCCTTCTCGTCCCCATGGATGACCCCCCTCAGATAGGGGTCCCTTGAC

T2CH39_PCRI ..............C....................................C......................................

T2CH39_PCRII ..............C....................................C......................................

T2CH39_I_1 ..............C....................................C......................................

T2CH39_I_2 ..............C....................................C......................................

T2CH39_I_3 ..............C....................................C......................................

T2CH39_I_5 ..............C....................................C......................................

T2CH39_I_6 ..............C....................................C......................................

T2CH39_I_7 ..............C....................A...............C......................................

T2CH39_I_8 ..............C....................................C......................................

T2CH39_I_9 ..............C....................................C......................................

T2CH39_I_10 ..............C....................................C......................................

T2CH39_II_1 ..............C....................................C......................................

T2CH39_II_2 ..............C..........................-------------------------------------------------

T2CH39_III_2 ..............C....................................C......................................

T2CH39_III_2 ..............C....................................C......................................

Sample T2CH61

PCR product from both DNA isolations (I,II) were cloned without direct sequencing, thus only sequences of clones are reported.

16120 16130 16140 16150 16160 16170 16180 16190 16200 16210

....|....|....|....|....|....|....|....|....|....|....|....|....|....|....|....|....|....|....|....|

CRS CCACCATGAATATTGTACGGTACCATAAATACTTGACCACCTGTAGTACATAAAAACCCAATCCACATCAAAACCCCCTCCCCATGCTTACAAGCAAGTA

T2CH61_I_7 -----------------------------------------------------------------------------.C..--.................

T2CH61_I_8 ........................................................................C.....C.....................

T2CH61_I_9 .........................................................T..............C.....C.....................

T2CH61_I_1 -----------------------------------------------------------------------------.C.....................

T2CH61_I_2 .........................................................T..............C.....C..--......G..........

T2CH61_I_3 .........................................................T..............C.....C...A-................

T2CH61_I_4 .........................................G...............T.......A.AT...C.....C.............G.......

T2CH61_I_5 .....................................................G...T..............C.....C.....................

T2CH61_II_1 ............................G............................T..............C.....C.....................

T2CH61_II_2 ----------------------------------------------------------------------------------------------------

T2CH61_II_5 ............................G............................T..............C.....C.....................

T2CH61_II_6 ----------------------------------------------------------------------------------------------------

T2CH61_II_7 .........................................................T..............C.....C.....................

T2CH61_II_8 .........................................................T..............C.....C..A--................

T2CH61_II_9 .........................................................T..............C.....C.....................

T2CH61_II_10 --------------------------------------------------------------------....C.....C.....................

T2CH61_II_11 --------------------------------------------------------------------------------..-.................

T2CH61_II_12 ...............A.............A............A..............T..............C.....C...-.................

Sample T2CH61 – continued

16220 16230 16240 16250 16260 16270 16280 16290 16300 16310

....|....|....|....|....|....|....|....|....|....|....|....|....|....|....|....|....|....|....|....|

CRS CAGCAATCAACCCTCAACTATCACACATCAACTGCAACTCCAAAGCCACCCCTCACCCACTAGGATACCAACAAACCTACCCACCCTTAACAGTACATAG

T2CH61_I_7 ......C.............................................................................T...............

T2CH61_I_8 ......C.......................................................................G.....................

T2CH61_I_9 ......C.............................................................................T...............

T2CH61_I_1 ......C.............................................................................T...............

T2CH61_I_2 ......C...........................................................T.................T...............

T2CH61_I_3 ......C..............................................................T..............T...............

T2CH61_I_4 ......C..........................A..................................................T...............

T2CH61_I_5 T.....C.............................................................................T...............

T2CH61_II_1 ......C.............................................................................T...............

T2CH61_II_2 ---------....................TT.....................................................T...............

T2CH61_II_5 ......C.............................................................................T...............

T2CH61_II_6 ---------------------...............................................................T...............

T2CH61_II_7 ......C.............................................................................T...............

T2CH61_II_8 ......C........G....................................................................T...............

T2CH61_II_9 ......C.............................................................................T...............

T2CH61_II_10 ......C.............................................................................T...............

T2CH61_II_11 ......C.............................................................................T...............

T2CH61_II_12 ......C.............................................................................T...............

16320 16330 16340 16350 16360 16370 16380 16390 16400

....|....|....|....|....|....|....|....|....|....|....|....|....|....|....|....|....|....|

CRS TACATAAAGCCATTTACCGTACATAGCACATTACAGTCAAATCCCTTCTCGTCCCCATGGATGACCCCCCTCAGATAGGGGTCCCTTGAC

T2CH61_I_7 ................................................C.........................................

T2CH61_I_8 ..........................................................................................

T2CH61_I_9 ............................T.............................................................

T2CH61_I_1 ..........................................................................................

T2CH61_I_2 ........................G.................................................................

T2CH61_I_3 .......................C.......................................G..........................

T2CH61_I_4 ......................................................................................C...

T2CH61_I_5 ..........................................................................................

T2CH61_II_1 ..........................................................................................

T2CH61_II_2 ........................................................................G.................

T2CH61_II_5 ..........................................................................................

T2CH61_II_6 ..........................................................................................

T2CH61_II_7 ...G.................................................................................C....

T2CH61_II_8 ..........................................................................................

T2CH61_II_9 ..........................................................................................

T2CH61_II_10 ..........................................................................................

T2CH61_II_11 .......................................G..................................................

T2CH61_II_12 ..........................................................................................

Sample T2CH71

PCR product from first DNA isolation (I) was cloned without direct sequencing, thus only sequences of clones are reported.

16120 16130 16140 16150 16160 16170 16180 16190 16200 16210

....|....|....|....|....|....|....|....|....|....|....|....|....|....|....|....|....|....|....|....|

CRS CCACCATGAATATTGTACGGTACCATAAATACTTGACCACCTGTAGTACATAAAAACCCAATCCACATCAAAACCCCCTCCCCATGCTTACAAGCAAGTA

T2CH71_PCRII ----------------------.........................G..............................C.....................

T2CH71_I_1 .................................G...........................................-C.....................

T2CH71_I_5 ---------------------------------------------------...........................C.....................

T2CH71_I_6 ----------------------------------------------------------------------------------..................

T2CH71_I_7 ..............................................................................C.....................

T2CH71_I_8 ..............................................................................C.....................

T2CH71_I_9 ------------------------------------------------------------------------------C.....................

T2CH71_I_10 ---...........................................................................C.....................

T2CH71_I_11 ..............................................................................C.....................

T2CH71_II_1 ..............................................................................C............C........

T2CH71_II_2 ..............................................................................C.....................

16220 16230 16240 16250 16260 16270 16280 16290 16300 16310

....|....|....|....|....|....|....|....|....|....|....|....|....|....|....|....|....|....|....|....|

CRS CAGCAATCAACCCTCAACTATCACACATCAACTGCAACTCCAAAGCCACCCCTCACCCACTAGGATACCAACAAACCTACCCACCCTTAACAGTACATAG

T2CH71_PCRII ............T..........................................................................C...........r

T2CH71_I_1 ............T..........................................................................C...........A

T2CH71_I_5 ............T..........................................................................C...........A

T2CH71_I_6 ............T..........................................................................C...........A

T2CH71_I_7 ............T..........................................................................C...........A

T2CH71_I_8 ............T..........................................................................C...........A

T2CH71_I_9 ............T..........................................................................C...........A

T2CH71_I_10 ............T..............................G...........................................C...........A

T2CH71_I_11 ............T..........................................................................C...........A

T2CH71_II_1 .C..........T..................................C.......................................C...........A

T2CH71_II_2 ............T...............................................C..........................C............

Sample T2CH71 – continued

16320 16330 16340 16350 16360 16370 16380 16390 16400

....|....|....|....|....|....|....|....|....|....|....|....|....|....|....|....|....|....|

CRS TACATAAAGCCATTTACCGTACATAGCACATTACAGTCAAATCCCTTCTCGTCCCCATGGATGACCCCCCTCAGATAGGGGTCCCTTGAC

T2CH71_PCRII ..............C.T..............................................................-----------

T2CH71_I_1 ..............C.T.........................................................................

T2CH71_I_5 ..............C.T...............................................................A.........

T2CH71_I_6 ..............C.T.........................................................................

T2CH71_I_7 ..............C.T.........................................................................

T2CH71_I_8 ..............C.T.........................................................................

T2CH71_I_9 ..............C.T.........................................................................

T2CH71_I_10 ..............C.T.........................................................................

T2CH71_I_11 ..............C.T.........................................................................

T2CH71_II_1 ..............C.T........................................................A.A............G.

T2CH71_II_2 ..............C.T.........................................................................

Sample T2CH72

16120 16130 16140 16150 16160 16170 16180 16190 16200 16210

....|....|....|....|....|....|....|....|....|....|....|....|....|....|....|....|....|....|....|....|

CRS CCACCATGAATATTGTACGGTACCATAAATACTTGACCACCTGTAGTACATAAAAACCCAATCCACATCAAAACCCCCTCCCCATGCTTACAAGCAAGTA

T2CH72_PCRI -----------........................................................c...YY.....Y.....................

T2CH72_PCRII ------------.......................................................C...CC.....c.....................

T2CH72_I_1 ...................................................................C...CC.....CT....................

T2CH72_I_2 ...................................................................C...CC.....CT....................

T2CH72_I_3 -------------------------------------------------------............C..GCC.....C.....................

T2CH72_I_4 -------------------------------------------------------G...........C...C-.....C.....................

T2CH72_I_5 -------------------------------------------------------............C...C-.....C.....................

T2CH72_I_6 -----------------------------------------------...A................C..........C.........G...........

T2CH72_II_1 ...............C....................................................................................

T2CH72_II_2 ...................................................................C...--.....C.....................

T2CH72_II_3 ...................................................................C...CC.....C.....................

16220 16230 16240 16250 16260 16270 16280 16290 16300 16310

....|....|....|....|....|....|....|....|....|....|....|....|....|....|....|....|....|....|....|....|

CRS CAGCAATCAACCCTCAACTATCACACATCAACTGCAACTCCAAAGCCACCCCTCACCCACTAGGATACCAACAAACCTACCCACCCTTAACAGTACATAG

T2CH72_PCRI ......Y.............................................................................................

T2CH72_PCRII ......C........................................G....................................................

T2CH72_I_1 ......C.....T...................................................................TT...T..............

T2CH72_I_2 ......C.....T...........................................T.......................TT...T..............

T2CH72_I_3 ......C.....G...GG................................T.................................................

T2CH72_I_4 ......C.............................................................................................

T2CH72_I_5 ......C.............................................................................................

T2CH72_I_6 ......C...........................................................................G.................

T2CH72_II_1 ..A.................................................................................................

T2CH72_II_2 ......C.............................................................................................

T2CH72_II_3 ......C......................................................G......................................

16320 16330 16340 16350 16360 16370 16380 16390 16400

....|....|....|....|....|....|....|....|....|....|....|....|....|....|....|....|....|....|

CRS TACATAAAGCCATTTACCGTACATAGCACATTACAGTCAAATCCCTTCTCGTCCCCATGGATGACCCCCCTCAGATAGGGGTCCCTTGAC

T2CH72_PCRI .............................................................................

T2CH72_PCRII .....................................................T.........C....T.AG.TT.G.TC

T2CH72_I_1 ..........................................T...............................................

T2CH72_I_2 ..........................................T...............................................

T2CH72_I_3 ...............................................................G..........................

T2CH72_I_4 ..........................................................................................

T2CH72_I_5 ..........................................................................................

T2CH72_I_6 ..........................................................................................

T2CH72_II_1 ..........................................................................................

T2CH72_II_2 .............................................................................AA...........

T2CH72_II_3 ..........................................................................................

Sample T2CH73

16120 16130 16140 16150 16160 16170 16180 16190 16200 16210

....|....|....|....|....|....|....|....|....|....|....|....|....|....|....|....|....|....|....|....|

CRS CCACCATGAATATTGTACGGTACCATAAATACTTGACCACCTGTAGTACATAAAAACCCAATCCACATCAAAACCCCCTCCCCATGCTTACAAGCAAGTA

T2CH73_PCRI -----------------..................................................C...CC.....C.....................

T2CH73_PCRII --------------.....................................................C...CC.....C.....................

T2CH73_I_1 ...................................................................C...CC.....C.....................

T2CH73_I_3 ...................................................................C...CC.....C.....................

T2CH73_I_5 ...................................................................C...CC.....C.....................

T2CH73_II_1 ...................................................................C...CC.....C.............G.......

T2CH73_III_1 -----------------------------------------------------------------------CC.....C.....................

T2CH73_III_2 ------------------------------------------------------------------------C.....C.....................

T2CH73_III_3 ------------------------------------------------------------------------C.....C.....................

16220 16230 16240 16250 16260 16270 16280 16290 16300 16310

....|....|....|....|....|....|....|....|....|....|....|....|....|....|....|....|....|....|....|....|

CRS CAGCAATCAACCCTCAACTATCACACATCAACTGCAACTCCAAAGCCACCCCTCACCCACTAGGATACCAACAAACCTACCCACCCTTAACAGTACATAG

T2CH73_PCRI ......C.............................................................................................

T2CH73_PCRII ......C.............................................................................................

T2CH73_I_1 ......C.............................................................................................

T2CH73_I_3 ......C.............................................................................................

T2CH73_I_5 ......C.............................................................................................

T2CH73_II_1 ......C.............................................................................................

T2CH73_III_1 ......C.............................................................................................

T2CH73_III_2 ......C.............................................................................................

T2CH73_III_3 ......C.............................................................................................

16320 16330 16340 16350 16360 16370 16380 16390 16400

....|....|....|....|....|....|....|....|....|....|....|....|....|....|....|....|....|....|

CRS TACATAAAGCCATTTACCGTACATAGCACATTACAGTCAAATCCCTTCTCGTCCCCATGGATGACCCCCCTCAGATAGGGGTCCCTTGAC

T2CH73_PCRI ........................................

T2CH73_PCRII ...........................................................................

T2CH73_I_1 ..........................................................................................

T2CH73_I_3 ..........................................................................................

T2CH73_I_5 .............................................................................A............

T2CH73_II_1 ....A...............................C.....................................................

T2CH73_III_1 ..........................................................................................

T2CH73_III_2 ...............-........................................................................G.

T2CH73_III_3 ...............-........................................................................G.

Sample T2CH711

16120 16130 16140 16150 16160 16170 16180 16190 16200 16210

....|....|....|....|....|....|....|....|....|....|....|....|....|....|....|....|....|....|....|....|

CRS CCACCATGAATATTGTACGGTACCATAAATACTTGACCACCTGTAGTACATAAAAACCCAATCCACATCAAAACCCCCTCCCCATGCTTACAAGCAAGTA

T2CH711_PCRI -----..................................................................CC.....C.....................

T2CH711-1I .......................................................................CC.....C.....................

T2CH711-2I .......A...............................................................CC.....C.....................

T2CH711-4I .......................................................................CC.....C...-.................

16220 16230 16240 16250 16260 16270 16280 16290 16300 16310

....|....|....|....|....|....|....|....|....|....|....|....|....|....|....|....|....|....|....|....|

CRS CAGCAATCAACCCTCAACTATCACACATCAACTGCAACTCCAAAGCCACCCCTCACCCACTAGGATACCAACAAACCTACCCACCCTTAACAGTACATAG

T2CH711_PCRI ......C.............................G.............T.................................................

T2CH711-1I ......C.............................G.............T.................................................

T2CH711-2I ......C.............................G.............T.................................................

T2CH711-4I ......C.............................G.............T.................................................

16320 16330 16340 16350 16360 16370 16380 16390 16400

....|....|....|....|....|....|....|....|....|....|....|....|....|....|....|....|....|....|

CRS TACATAAAGCCATTTACCGTACATAGCACATTACAGTCAAATCCCTTCTCGTCCCCATGGATGACCCCCCTCAGATAGGGGTCCCTTGAC

T2CH711_PCRI ....................................................................................------

T2CH711-1I ..........................................................................................

T2CH711-2I ..........................................................................................

T2CH711-4I .................................................................................G........

Sample T2CH712

PCR product from both DNA isolations (I,II) were cloned without direct sequencing, thus only sequences of clones are reported.

16120 16130 16140 16150 16160 16170 16180 16190 16200 16210

....|....|....|....|....|....|....|....|....|....|....|....|....|....|....|....|....|....|....|....|

CRS CCACCATGAATATTGTACGGTACCATAAATACTTGACCACCTGTAGTACATAAAAACCCAATCCACATCAAAACCCCCTCCCCATGCTTACAAGCAAGTA

T2CH712_I_1 .......................................................................CC.....C.....................

T2CH712_I_5 ......................................G................................CC.....C...-.................

T2CH712_I_2 ................................C......................................CC.....C..--.................

T2CH712_I_4 .......................................................................CC.....C...-.................

T2CH712_II_1 ......................................G................................-C.-...C.....................

T2CH712_II_2 .......................................................................CC.....C.....................

16220 16230 16240 16250 16260 16270 16280 16290 16300 16310

....|....|....|....|....|....|....|....|....|....|....|....|....|....|....|....|....|....|....|....|

CRS CAGCAATCAACCCTCAACTATCACACATCAACTGCAACTCCAAAGCCACCCCTCACCCACTAGGATACCAACAAACCTACCCACCCTTAACAGTACATAG

T2CH712_I_1 ......C.............................G.............T.................................................

T2CH712_I_5 ......C.............................G.............T.................................................

T2CH712_I_2 ......C.................G...........G.............T.................................................

T2CH712_I_4 ......C.............................T.............T.................................................

T2CH712_II_1 ......C.............................G.............T............-------------------------------------

T2CH712_II_2 ......C.............................G.............T.................................................

16320 16330 16340 16350 16360 16370 16380 16390 16400

....|....|....|....|....|....|....|....|....|....|....|....|....|....|....|....|....|....|

CRS TACATAAAGCCATTTACCGTACATAGCACATTACAGTCAAATCCCTTCTCGTCCCCATGGATGACCCCCCTCAGATAGGGGTCCCTTGAC

T2CH712_I_1 ..........................................................................................

T2CH712_I_5 ...G......................................................................................

T2CH712_I_2 ..........................................................................................

T2CH712_I_4 ..........................................................................................

T2CH712_II_1 ------------------------------------------------------------------------------------------

T2CH712_II_2 ..........................................................................................

Sample T2CH715

16120 16130 16140 16150 16160 16170 16180 16190 16200 16210

....|....|....|....|....|....|....|....|....|....|....|....|....|....|....|....|....|....|....|....|

CRS CCACCATGAATATTGTACGGTACCATAAATACTTGACCACCTGTAGTACATAAAAACCCAATCCACATCAAAACCCCCTCCCCATGCTTACAAGCAAGTA

T2CH715_PCRII ------------------.......................................T..............C.....C.....................

T2CH715_PCRI ----------...............................................T..............C.....C.....................

T2CH715_I_1 ------------------------------------------------------...T..............C.....C.....................

T2CH715_I_6 .........................................................T..............C.....C.................G...

T2CH715_I_3 ................G........................................T..............C.....C--...................

T2CH715_I_2 .........................................................T..............C.....C---..................

T2CH715_I_5 .........................................................T..............C.....C.-......C............

T2CH715_I_4 .........................................................T..............C.....C.....................

16220 16230 16240 16250 16260 16270 16280 16290 16300 16310

....|....|....|....|....|....|....|....|....|....|....|....|....|....|....|....|....|....|....|....|

CRS CAGCAATCAACCCTCAACTATCACACATCAACTGCAACTCCAAAGCCACCCCTCACCCACTAGGATACCAACAAACCTACCCACCCTTAACAGTACATAG

T2CH715_PCRII ......C.............................................................................T...............

T2CH715_PCRI ......C.............................................................................T...............

T2CH715_I_1 ......C.............................................................................T...............

T2CH715_I_6 ......C.............................................................................T...............

T2CH715_I_3 ......C.............................................................................T...............

T2CH715_I_2 ......C.............................................................................T...............

T2CH715_I_5 ......C.............................................................................T...............

T2CH715_I_4 ......C.............................................................................T...............

16320 16330 16340 16350 16360 16370 16380 16390 16400

....|....|....|....|....|....|....|....|....|....|....|....|....|....|....|....|....|....|

CRS TACATAAAGCCATTTACCGTACATAGCACATTACAGTCAAATCCCTTCTCGTCCCCATGGATGACCCCCCTCAGATAGGGGTCCCTTGAC

T2CH715_PCRII .........................................................................-----------------

T2CH715_PCRI ...........................................................................---------------

T2CH715_I_1 ..........................................................................................

T2CH715_I_6 ..........................................................................................

T2CH715_I_3 ..........................................................................................

T2CH715_I_2 ..........................................................................................

T2CH715_I_5 ..........................................................................................

T2CH715_I_4 ................................................A.........................................

Sample T2CH719

16120 16130 16140 16150 16160 16170 16180 16190 16200 16210

....|....|....|....|....|....|....|....|....|....|....|....|....|....|....|....|....|....|....|....|

CRS CCACCATGAATATTGTACGGTACCATAAATACTTGACCACCTGTAGTACATAAAAACCCAATCCACATCAAAACCCCCTCCCCATGCTTACAAGCAAGTA

T2CH719_PCRI ....................................................................................................

T2CH719_PCRII ....................................................................................................

T2CH719-1I ....................................................................................................

T2CH719-2I ................................C...................................................................

T2CH719-3I ....................................................................................................

T2CH719-4I ....................................................................................................

T2CH719-5I ...........................................C........................................................

T2CH719-6I ....................................................................................................

T2CH719-1II ....................................................................................................

T2CH719-2II -------------------------------.....................................................................

16220 16230 16240 16250 16260 16270 16280 16290 16300 16310

....|....|....|....|....|....|....|....|....|....|....|....|....|....|....|....|....|....|....|....|

CRS CAGCAATCAACCCTCAACTATCACACATCAACTGCAACTCCAAAGCCACCCCTCACCCACTAGGATACCAACAAACCTACCCACCCTTAACAGTACATAG

T2CH719_PCRI ............T.......................................................................................

T2CH719_PCRII ............T.......................................................................................

T2CH719-1I ............T.......................................................................................

T2CH719-2I ....G.......T.......................................................................................

T2CH719-3I ............T.......................................................................................

T2CH719-4I .........G..T....T..................................................................................

T2CH719-5I ........G...T.......................................................................................

T2CH719-6I ............T.......................................................................................

T2CH719-1II ............T.......................................................................................

T2CH719-2II ............T.......................................................................................

16320 16330 16340 16350 16360 16370 16380 16390 16400

....|....|....|....|....|....|....|....|....|....|....|....|....|....|....|....|....|....|

CRS TACATAAAGCCATTTACCGTACATAGCACATTACAGTCAAATCCCTTCTCGTCCCCATGGATGACCCCCCTCAGATAGGGGTCCCTTGAC

T2CH719_PCRI ..............C....................................C......................................

T2CH719_PCRII ..............C....................................C......................................

T2CH719-1I ...G..........C....................................C......................................

T2CH719-2I ..............C...........................T........C......................................

T2CH719-3I ..............C....................................C......................................

T2CH719-4I ..............C....................................C......................................

T2CH719-5I ..............C........................G...........C......................................

T2CH719-6I ..............C....................................C............T.........................

T2CH719-1II ..............C....................................C......................................

T2CH719-2II ..............C....................................C......................................

Sample T2CH728

PCR product from first DNA isolation (I) was cloned without direct sequencing, thus only sequences of clones are reported.

16120 16130 16140 16150 16160 16170 16180 16190 16200 16210

....|....|....|....|....|....|....|....|....|....|....|....|....|....|....|....|....|....|....|....|

CRS CCACCATGAATATTGTACGGTACCATAAATACTTGACCACCTGTAGTACATAAAAACCCAATCCACATCAAAACCCCCTCCCCATGCTTACAAGCAAGTA

T2CH728_PCRII ---------..........................................................C...CC.....C.....................

T2CH728_I_2 ........................G..........................................C...CC.....C.....................

T2CH728_I_5 ...................................................................C...CC.....C.....................

T2CH728_I_10 ...................................................................C...CC.....C.....................

T2CH728_II_1 ...................................................................C...CC.....C.....................

T2CH728_II_2 -----------------------------------------..........................C...CC.....C.....................

16220 16230 16240 16250 16260 16270 16280 16290 16300 16310

....|....|....|....|....|....|....|....|....|....|....|....|....|....|....|....|....|....|....|....|

CRS CAGCAATCAACCCTCAACTATCACACATCAACTGCAACTCCAAAGCCACCCCTCACCCACTAGGATACCAACAAACCTACCCACCCTTAACAGTACATAG

T2CH728_PCRII ......CT............................................................................................

T2CH728_I_2 ......CT............................................................................................

T2CH728_I_5 ......CT............................................................................................

T2CH728_I_10 ......CT............................................................................................

T2CH728_II_1 ......CT......................G.....................................................................

T2CH728_II_2 ......C.............................................................................................

16320 16330 16340 16350 16360 16370 16380 16390 16400

....|....|....|....|....|....|....|....|....|....|....|....|....|....|....|....|....|....|

CRS TACATAAAGCCATTTACCGTACATAGCACATTACAGTCAAATCCCTTCTCGTCCCCATGGATGACCCCCCTCAGATAGGGGTCCCTTGAC

T2CH728_PCRII ..................................................................................--------

T2CH728_I_2 ..........................................................................................

T2CH728_I_5 ...............................................................G..........................

T2CH728_I_10 ..........................................................................................

T2CH728_II_1 ..........................................................................................

T2CH728_II_2 ........................................................................------------------

Sample T2CH729

PCR product from first DNA isolation (I) was cloned without direct sequencing, thus only sequences of clones are reported.

16120 16130 16140 16150 16160 16170 16180 16190 16200 16210

....|....|....|....|....|....|....|....|....|....|....|....|....|....|....|....|....|....|....|....|

CRS CCACCATGAATATTGTACGGTACCATAAATACTTGACCACCTGTAGTACATAAAAACCCAATCCACATCAAAACCCCCTCCCCATGCTTACAAGCAAGTA

T2CH729_PCRII ....................................................................................................

T2CH729_I_1 .......................T............................................................................

T2CH729_I_3 ....................................................................................................

T2CH729_I_4 ............................................................................T.......................

T2CH729_I_5 ................................................T.......T..........................G................

T2CH729_II_1 ....................................................................................................

T2CH729_II_2 ....................................................................................................

16220 16230 16240 16250 16260 16270 16280 16290 16300 16310

....|....|....|....|....|....|....|....|....|....|....|....|....|....|....|....|....|....|....|....|

CRS CAGCAATCAACCCTCAACTATCACACATCAACTGCAACTCCAAAGCCACCCCTCACCCACTAGGATACCAACAAACCTACCCACCCTTAACAGTACATAG

T2CH729_PCRII ............T.......................................................................................

T2CH729_I_1 ............T...............T.......................................................................

T2CH729_I_3 ............T.....C.................................................................................

T2CH729_I_4 ............T.......................................................................................

T2CH729_I_5 ...T........T.......................................................................................

T2CH729_II_1 ............T.......................................................................................

T2CH729_II_2 ............T.......................................................................................

16320 16330 16340 16350 16360 16370 16380 16390 16400

....|....|....|....|....|....|....|....|....|....|....|....|....|....|....|....|....|....|

CRS TACATAAAGCCATTTACCGTACATAGCACATTACAGTCAAATCCCTTCTCGTCCCCATGGATGACCCCCCTCAGATAGGGGTCCCTTGAC

T2CH729_PCRII ..............C....................................C......................................

T2CH729_I_1 ..............C....................................C......................................

T2CH729_I_3 ..............C....................................C......................................

T2CH729_I_4 ..............C....................................C......................................

T2CH729_I_5 ..............C.............T......................C......................................

T2CH729_II_1 ..............C....................................C......................................

T2CH729_II_2 ..............C....................................C......................................

Sample T2CH730

16120 16130 16140 16150 16160 16170 16180 16190 16200 16210

....|....|....|....|....|....|....|....|....|....|....|....|....|....|....|....|....|....|....|....|

CRS CCACCATGAATATTGTACGGTACCATAAATACTTGACCACCTGTAGTACATAAAAACCCAATCCACATCAAAACCCCCTCCCCATGCTTACAAGCAAGTA

T2CH730_PCRI T...................................................................................................

T2CH730_PCRII T...................................................................................................

T2CH730_I_1 T...................................................................................................

T2CH730_I_5 T...................................................................................................

T2CH730_I_4 T...................................................................................................

T2CH730_I_6 T..........................................................................T........................

T2CH730_II_1 T...................................................................................................

T2CH730_II_2 T...................................................................................................

16220 16230 16240 16250 16260 16270 16280 16290 16300 16310

....|....|....|....|....|....|....|....|....|....|....|....|....|....|....|....|....|....|....|....|

CRS CAGCAATCAACCCTCAACTATCACACATCAACTGCAACTCCAAAGCCACCCCTCACCCACTAGGATACCAACAAACCTACCCACCCTTAACAGTACATAG

T2CH730_PCRI ......C.....T..................................................................T....................

T2CH730_PCRII ......C.....T..................................................................T....................

T2CH730_I_1 ......C.....T.T................................................................T....................

T2CH730_I_5 ......C.....T..................................................................T....................

T2CH730_I_4 ......C.....T..................................................................T....................

T2CH730_I_6 ......C.....T..................................................................T....................

T2CH730_II_1 ......C.....T..................................................................T....................

T2CH730_II_2 ......C.....T..................................................................T....................

16320 16330 16340 16350 16360 16370 16380 16390 16400

....|....|....|....|....|....|....|....|....|....|....|....|....|....|....|....|....|....|

CRS TACATAAAGCCATTTACCGTACATAGCACATTACAGTCAAATCCCTTCTCGTCCCCATGGATGACCCCCCTCAGATAGGGGTCCCTTGAC

T2CH730_PCRI ........A..........................................C......................................

T2CH730_PCRII ........A..........................................C......................................

T2CH730_I_1 ........A..........................................C......................................

T2CH730_I_5 ........A...........................C..............C......................................

T2CH730_I_4 ........A..........................................C..T.............T.....................

T2CH730_I_6 ........A..........................................C......................................

T2CH730_II_1 ........A..........................................C......................................

T2CH730_II_2 ........A..........................................C......................................

Sample T2CH82

16120 16130 16140 16150 16160 16170 16180 16190 16200 16210

....|....|....|....|....|....|....|....|....|....|....|....|....|....|....|....|....|....|....|....|

CRS CCACCATGAATATTGTACGGTACCATAAATACTTGACCACCTGTAGTACATAAAAACCCAATCCACATCAAAACCCCCTCCCCATGCTTACAAGCAAGTA

T2CH82_PCRI -----------.............................................................C.....C.....................

T2CH82_PCRII -----------------.......................................................C.....C.....................

T2CH82_I_1 ........................................................................C.....C.....................

T2CH82_II_1 -------------------------------------------------------.................C.....C.....................

T2CH82_II_2 ........................................................................C.....C.....................

T2CH82_II_3 ........................................................................C.....C.....................

16220 16230 16240 16250 16260 16270 16280 16290 16300 16310

....|....|....|....|....|....|....|....|....|....|....|....|....|....|....|....|....|....|....|....|

CRS CAGCAATCAACCCTCAACTATCACACATCAACTGCAACTCCAAAGCCACCCCTCACCCACTAGGATACCAACAAACCTACCCACCCTTAACAGTACATAG

T2CH82_PCRI ......C.......................................................................G.....................

T2CH82_PCRII ......C.......................................................................G.....................

T2CH82_I_1 ......C.......................................................................G.....................

T2CH82_II_1 ......C.......................................................................G.....................

T2CH82_II_2 ......C.......................................................................G.....................

T2CH82_II_3 ......C......................................................G................G.....................

16320 16330 16340 16350 16360 16370 16380 16390 16400

....|....|....|....|....|....|....|....|....|....|....|....|....|....|....|....|....|....|

CRS TACATAAAGCCATTTACCGTACATAGCACATTACAGTCAAATCCCTTCTCGTCCCCATGGATGACCCCCCTCAGATAGGGGTCCCTTGAC

T2CH82_PCRI .............................................................................-------------

T2CH82_PCRII ...............................................................................-----------

T2CH82_I_1 ..........................................................................................

T2CH82_II_1 ..........................................................................................

T2CH82_II_2 ..........................................................................................

T2CH82_II_3 ..........................................................................................

Sample T2CH83

PCR product from both DNA isolations (I,II) were cloned without direct sequencing, thus only sequences of clones are reported.

16120 16130 16140 16150 16160 16170 16180 16190 16200 16210

....|....|....|....|....|....|....|....|....|....|....|....|....|....|....|....|....|....|....|....|

CRS CCACCATGAATATTGTACGGTACCATAAATACTTGACCACCTGTAGTACATAAAAACCCAATCCACATCAAAACCCCCTCCCCATGCTTACAAGCAAGTA

T2CH83_I_1 .....................................T...................T..............C.....C.....................

T2CH83_I_2 .........................................................T..............C.....C.....................

T2CH83_I_2 .........................................................T..............C.....C.....................

T2CH83_I_4 .........................................................T..............C.....C.....................

T2CH83_I_5 .........................................................T..............C.....C.....................

T2CH83_I_6 .........................................................T..............C.....C.....................

T2CH83_I_7 ...............C....................................................................................

T2CH83_I_8 .........................................................T..............C.....C.....................

T2CH83_I_9 .........................................................T..............C.....C.....................

T2CH83_I_10 .........................................................T..............C.....C.....................

T2CH83_II_1 ..................C......................................T..............C.....C.....................

T2CH83_II_2 .........................................................T..............C.....C.....................

T2CH83_II_3 ........................................................................C.....C.....................

16220 16230 16240 16250 16260 16270 16280 16290 16300 16310

....|....|....|....|....|....|....|....|....|....|....|....|....|....|....|....|....|....|....|....|

CRS CAGCAATCAACCCTCAACTATCACACATCAACTGCAACTCCAAAGCCACCCCTCACCCACTAGGATACCAACAAACCTACCCACCCTTAACAGTACATAG

T2CH83_I_1 ......C.............................................................................................

T2CH83_I_2 ......C.............................................................................................

T2CH83_I_2 ......C.............................................................................................

T2CH83_I_4 ......C.............................................................................................

T2CH83_I_5 ......C.............................................................................................

T2CH83_I_6 ......C.............................................................................................

T2CH83_I_7 ..A.................................................................................................

T2CH83_I_8 ......C...................................................................G.........................

T2CH83_I_9 ......C.............................................................................................

T2CH83_I_10 ......C.............................................................................................

T2CH83_II_1 ......C...................................G.........................................................

T2CH83_II_2 ......C.................................T...........................................................

T2CH83_II_3 ......C.......................................................................G.....................

Sample T2CH83 – continued

16320 16330 16340 16350 16360 16370 16380 16390 16400

....|....|....|....|....|....|....|....|....|....|....|....|....|....|....|....|....|....|

CRS TACATAAAGCCATTTACCGTACATAGCACATTACAGTCAAATCCCTTCTCGTCCCCATGGATGACCCCCCTCAGATAGGGGTCCCTTGAC

T2CH83_I_1 ...........G...................................................................A..........

T2CH83_I_2 ...............................................................................A..........

T2CH83_I_2 ...............................................................................A..........

T2CH83_I_4 ...............................................................................A..........

T2CH83_I_5 .....................T.........................................................A..........

T2CH83_I_6 ...............................................................................A..........

T2CH83_I_7 ...............................................................................A..........

T2CH83_I_8 ...............................................................................A..........

T2CH83_I_9 ................................................C..............................A..........

T2CH83_I_10 ...............................................................................A..........

T2CH83_II_1 ...............................................................................A..........

T2CH83_II_2 ...............................................................................A..........

T2CH83_II_3 ..........................................................................................

Sample T2CH84

16120 16130 16140 16150 16160 16170 16180 16190 16200 16210

....|....|....|....|....|....|....|....|....|....|....|....|....|....|....|....|....|....|....|....|

CRS CCACCATGAATATTGTACGGTACCATAAATACTTGACCACCTGTAGTACATAAAAACCCAATCCACATCAAAACCCCCTCCCCATGCTTACAAGCAAGTA

T2CH84_PCRI ------------------.......................................T..............C.....C.....................

T2CH84_PCRII --------------...........................................T..............C.....C.....................

T2CH84_I_1 ----------------------------------------------------------------------CCC.....C.....................

T2CH84_I_2 ---------------------------------------------------------------------..CC.....C.....................

T2CH84_I_3 ------------------------------------------------------------------------C.....C.....................

T2CH84_I_4 .........................................................T..............-.....C.....................

T2CH84_I_5 .........................................................T..............-.....C.T...................

T2CH84_I_6 .........................................................T..............-.....C.T...................

T2CH84_I_7 .........................................................T..............-.....C.T...................

T2CH84_II_1 .........................................................T..............-.....C.....................

T2CH84_III_1 -----------------------------............................T..............-.....C.....................

16220 16230 16240 16250 16260 16270 16280 16290 16300 16310

....|....|....|....|....|....|....|....|....|....|....|....|....|....|....|....|....|....|....|....|

CRS CAGCAATCAACCCTCAACTATCACACATCAACTGCAACTCCAAAGCCACCCCTCACCCACTAGGATACCAACAAACCTACCCACCCTTAACAGTACATAG

T2CH84_PCRI ......C.............................................................................T...............

T2CH84_PCRII ......C.............................................................................T...............

T2CH84_I_1 ......C.........................................................................T...T........C......

T2CH84_I_2 ......C.............................................................................T...............

T2CH84_I_3 ......C.............................................................................T...............

T2CH84_I_4 ......C.............................................................................T...............

T2CH84_I_5 ......C.............................................................................T...............

T2CH84_I_6 ......C.............................................................................T...............

T2CH84_I_7 ......C.............................................................................T...............

T2CH84_II_1 ......C.............................................................................T...............

T2CH84_III_1 ......C.............................................................................T...............

Sample T2CH84 – continued

16320 16330 16340 16350 16360 16370 16380 16390 16400

....|....|....|....|....|....|....|....|....|....|....|....|....|....|....|....|....|....|

CRS TACATAAAGCCATTTACCGTACATAGCACATTACAGTCAAATCCCTTCTCGTCCCCATGGATGACCCCCCTCAGATAGGGGTCCCTTGAC

T2CH84_PCRI ..................................................................

T2CH84_PCRII ................................................................

T2CH84_I_1 ..........................................................................................

T2CH84_I_2 ...............................C..........................................................

T2CH84_I_3 .....................................................................T....................

T2CH84_I_4 ........................G.................................................................

T2CH84_I_5 ..........................................................................................

T2CH84_I_6 ..........................................................................................

T2CH84_I_7 ..........................................................................................

T2CH84_II_1 ..........................................................................................

T2CH84_III_1 ........................................-......................---------------------------

Sample T2CH85

16120 16130 16140 16150 16160 16170 16180 16190 16200 16210

....|....|....|....|....|....|....|....|....|....|....|....|....|....|....|....|....|....|....|...

CRS CCACCATGAATATTGTACGGTACCATAAATACTTGACCACCTGTAGTACATAAAAACCCAATCCACATCAAAACCCCCTCCCCATGCTTACAAGCAAGTA

T2CH85_PCRI -----------..............................................T..............C.....C.....................

T2CH85_PCRII -----------..............................................T..............C.....C.....................

T2CH85_I_1 .........................................................T..............C.....C.....................

T2CH85_I_3 .........................................................T..............C.....C.....................

T2CH85_I_5 .........................................................T..............C.....C.....................

T2CH85_II_1 ----------------------------------------------------------------------------..C.A....T..C...........

T2CH85_II_2 ---------------------------------------..................T..............C.....C....-----------------

T2CH85_III_1 ............................G............................T..............C.....C.....................

T2CH85_III_5 ............................G............................T..............C.....C.....................

T2CH85_III_7 .........................................................T..............C.....C.....................

T2CH85_III_8 .........................................................T..............C.....C..--.................

T2CH85_III_9 .........................................................T..............C.....C.....................

16220 16230 16240 16250 16260 16270 16280 16290 16300 16310

....|....|....|....|....|....|....|....|....|....|....|....|....|....|....|....|....|....|....|....|

CRS CAGCAATCAACCCTCAACTATCACACATCAACTGCAACTCCAAAGCCACCCCTCACCCACTAGGATACCAACAAACCTACCCACCCTTAACAGTACATAG

T2CH85_PCRI ......C.............................................................................T...............

T2CH85_PCRII ......C.............................................................................T...............

T2CH85_I_1 ......C.............................................................................T...............

T2CH85_I_3 ......C....T........................................................................TT..............

T2CH85_I_5 ......C.............................................................................T...............

T2CH85_II_1 ......C.............................................................................T...............

T2CH85_II_2 ----------------------------------------------------------------------------------------------------

T2CH85_III_1 ......C.............................................................................T...............

T2CH85_III_5 ......C.............................................................................T...............

T2CH85_III_7 ......C.............................................................................T...............

T2CH85_III_8 ......C........G....................................................................T...............

T2CH85_III_9 ......C.............................................................................T...............

Sample T2CH85 – continued

16320 16330 16340 16350 16360 16370 16380 16390 16400

....|....|....|....|....|....|....|....|....|....|....|....|....|....|....|....|....|....|

CRS TACATAAAGCCATTTACCGTACATAGCACATTACAGTCAAATCCCTTCTCGTCCCCATGGATGACCCCCCTCAGATAGGGGTCCCTTGAC

T2CH85_PCRI ...................................................................................-------

T2CH85_PCRII ...................................................................................-------

T2CH85_I_1 ..........................................................................................

T2CH85_I_3 ..........................................................................................

T2CH85_I_5 ..........................................................................................

T2CH85_II_1 ............................................T...------------------------------------------

T2CH85_II_2 ------------------------------------------------------------------------------------------

T2CH85_III_1 ..........................................................................................

T2CH85_III_5 ..........................................................................................

T2CH85_III_7 ...G.................................................................................C....

T2CH85_III_8 ..........................................................................................

T2CH85_III_9 ..........................................................................................

Sample T2CH86

16120 16130 16140 16150 16160 16170 16180 16190 16200 16210

....|....|....|....|....|....|....|....|....|....|....|....|....|....|....|....|....|....|....|....|

CRS CCACCATGAATATTGTACGGTACCATAAATACTTGACCACCTGTAGTACATAAAAACCCAATCCACATCAAAACCCCCTCCCCATGCTTACAAGCAAGTA

T2CH86_PCRII ----------...............................................T..............C.....C.....................

T2CH86_PCRI -----------..............................................T..............C.....C.....................

T2CH86_I_1 .........................................................T..............C.....C.....................

T2CH86_I_2 ------------------------------------------------------------------------C.....C.....................

T2CH86_I_6 .........................................................T..............C.....C.....................

T2CH86_II_1 .........................................................T..............C.....C.....................

T2CH86_II_2 .........................................................T..............C.....C.....................

T2CH86_III_1 ---.......-..............................................T..............C.....C.....................

16220 16230 16240 16250 16260 16270 16280 16290 16300 16310

....|....|....|....|....|....|....|....|....|....|....|....|....|....|....|....|....|....|....|....|

CRS CAGCAATCAACCCTCAACTATCACACATCAACTGCAACTCCAAAGCCACCCCTCACCCACTAGGATACCAACAAACCTACCCACCCTTAACAGTACATAG

T2CH86_PCRII ......C.............................................................................T...............

T2CH86_PCRI ......C.............................................................................T...............

T2CH86_I_1 ......C..................T..........................................................T...............

T2CH86_I_2 ......C.............................................................................T...............

T2CH86_I_6 ......C.............................................................................T...............

T2CH86_II_1 ......C.............................................................................T...............

T2CH86_II_2 ......C.............................................................................T...............

T2CH86_III_1 ......C.............................................................................T...............

16320 16330 16340 16350 16360 16370 16380 16390 16400

....|....|....|....|....|....|....|....|....|....|....|....|....|....|....|....|....|....|

CRS TACATAAAGCCATTTACCGTACATAGCACATTACAGTCAAATCCCTTCTCGTCCCCATGGATGACCCCCCTCAGATAGGGGTCCCTTGAC

T2CH86_PCRII ..............................................................................------------

T2CH86_PCRI .................................................................................---------

T2CH86_I_1 ..........................................................................................

T2CH86_I_2 ..........................................................................................

T2CH86_I_6 ..........................................................................................

T2CH86_II_1 ..........................................................................................

T2CH86_II_2 ..........................................................................................

T2CH86_III_1 ......................................................................--------------------
