# Supplementary material for: Ancient DNA reveals kinship burial patterns of a pre-Columbian Andean community
Source: BMC Genet. 2012 Apr 23;13:30. doi: 10.1186/1471-2156-13-30 (PMC3470988; doi:10.1186/1471-2156-13-30)
Supplement: Additional file 3 — Details of Y chromosome STR multiplexes design and validation. [file 1471-2156-13-30-S3.doc]

**Additional file 3.** Details of Y chromosome STR multiplexes design and validation.

Analysis of nuclear DNA from ancient samples is limited to these of extraordinary preservation. During analysis of the Y-chromosome STR profiles obtained with AmpFlSTR Y Filler kit (AB) loci and allele drop-outs were observed, especially for loci with amplicons longer than 200 bp. For example for locus DYS392 (292 – 325 bp). Due to smaler discrimination power of Y chromosome STRs collecting additional data were necessary. To amplify missing alleles a set of new multiplexes were designed accordingly to previously published guidelines [1-3]. Three new multiplexes covered all loci amplified with Y Filler besides H4 locus. Amplicons lengths were kept as short as possible. New starters were designed with Primer3 software and were checked for interactions and secondary structures with Primer Dimer and FastPCR software (Table S1). Allelic ladders were constructed according to Shoske et al., 2003 [2] from those supplied with AmpFlSTR Y Filler kit. To ensure reliability of new multiplexes concordance study between new primer sets and AmpFlSTR Y Filler kit were conducted with 10 random contemporary male samples and 9945 control template (Applied Biosystems) resulting in complete repeatibility of typing. To estimate reactions sensitivity we perform reactions on a set of serial 10-fold dilutions of control template (100, 80, 50, 10, 5 pg of genomic DNA). Full and corect profiles were obtained with 100 and 80 pg of genomic DNA and up to 50% of correct allele calls were obtained with as low as 50 pg of genomic DNA.

**Table S1**. Primer sequences size ranges and concentrations of newly designed Y chromosome multiplexes.

| Locus | Primer sequence and dye | Size range (bp) | Final concentration | Ref. |
| --- | --- | --- | --- | --- |
| MultiPlex Y1 | | | | |
| DYS19F | 5'-Hex-ACTGAGTTTCTGTTATAGTGTTTT-3' | 140-176 | 0,3μM | [2] |
| DYS19R | 5'- GGTTAAGGAGAGTGTCACTA -3' |  | 0,3μM | This study |
| DYS635F | 5'-Tamra-GGCTTCTCACTTTGCATAGAATC -3' | 147-179 | 0,3μM | This study |
| DYS635R | 5'-GACCAGACCCAAATATCCATCA-3' |  | 0,3μM | This study |
| DYS392F | 5'-Fam-AGCCAAGAAGGAAAACAAA-3' | 88-124 | 0,3μM | [2] |
| DYS392R | 5'-GCCTACCAATCCCATTCCTTAG-3' |  | 0,3μM | This study |
| DYS393F | 5'-Tamra-GTGGTCTTCTACTTGTGTCAATAC-3' | 101-141 | 0,3μM | [2] |
| DYS393R | 5'-GAACTCAAGTCCAAAAAATGAGG-3' |  | 0,3μM | [2] |
| DYS437F | 5'-Hex-GGACTATGGGCGTGAGTGC-3' | 119-135 | 0,1μM | This study |
| DYS437R | 5'-GATAAGTAGATAGACATCATTCACAGA-3' |  | 0,1μM | This study |
| DYS438F | 5'-Fam-TGGGGAATAGTTGAACGGTAA-3’ | 137-179 | 0,2μM | [4] |
| DYS438R | 5'-GGAGGTTGTGGTGAGTCGAG-3' |  | 0,2μM | This study |
| DYS448F | 5'-Fam-GAAAGGGAGATAGAGACATGGA-3' | 190-232 | 0,2μM | This study |
| DYS448R | 5'-GTGGCCGGTCTGGAAATTTAT-3' |  | 0,2μM | This study |
| DYS456F | 5'-Hex-GGACCTTGTGATAATGTAAGATAG-3' | 89-113 | 0,1μM | [2] |
| DYS456R | 5'-GTAGGGACAGAACTAATGGAA-3' |  | 0,1μM | [2] |
| MultiPlex Y2 | | | | |
| DYS385F | 5'-Tamra-GAAGGAAGGAAGGAAGGGAAA-3' | 85-165 | 0,3μM | Park et al. (2007) |
| DYS385R | 5'-GTCTATCTATTCCAATTACATAGTCCTC-3' |  | 0,3μM | This study |
| DYS439F | 5'-Fam-GGTGGAGACAGATAGATGATAA-3' | 88-120 | 0,1μM | This study |
| DYS439R | 5'-GACCCATCATCTCTTTACTTATACT-3' |  | 0,1μM | This study |
| DYS390F | 5'-Hex- CTGCATTTTGGTACCCCATA-3’ | 144-192 | 0,1μM | Park et al. (2007) |
| DYS390R | 5'- GCAATGTGTATACTCAGAAACAAGG-3’ |  | 0,1μM | Park et al. (2007) |
| MultiPlex Y3 | | | | |
| DYS458F | 5'-Fam-GCAACAGGAATGAAACTCCAA -3' | 154-186 | 0,2μM | This study |
| DYS458R | 5'-GTTTCCTGACCTTGTGATCCAG-3' |  | 0,2μM | This study |
| DYS389F | 5'-Fam-CCAACTCTCATCTGTATTATCTATG-3' | 121-149 | 0,2μM | Shoske et al. (2003) |
| DYS389R | 5'-GAGATAGATGATGGACTGCTAGA-3' | 233-275 | 0,2μM | This study |
| DYS391F | 5'-Hex-TTCAATCATACACCCATATCTGTC-3' | 85-121 | 0,2μM | Shoske et al. (2003) |
| DYS391R | 5'-GATAGAGGGATAGGTAGGCAGGC-3' |  | 0,2μM | Shoske et al. (2003) |

**Additional references**

1. Wiegand P and Kleiber M: **Less is more – lenght reduction of STR amplicons using redesigned primers.** *International Journal of Legal Medicine* 2006, **120**:160-4.

2. Shoske R, Vallone PM, Ruitberg M et al.: **The design, optimalisation and testing of Y chromosome short tandem repeats (STR) loci.** *Analitical and Bioanalytical Chemistry* 2003, **375**:333-43.

3. Park MJ, Lee HY, Chung U et al.: **Y-STR analysis of degraded DNA using reduced-size amplikons.** *International Journal of Legal Medicine* 2007, **121**:152-7.

4. Ayub Q, Mohyuddin A, Qamar R et al.: **Identification and characterisation of novel human Y chromosomal microsatellites from sequence database information.** *Nucleic Acids Research* 2000, **28**:e8.
